# Interacting Effects of Newcastle Disease Transmission and Illegal Trade on a Wild Population of White-Winged Parakeets in Peru: A Modeling Approach

**DOI:** 10.1371/journal.pone.0147517

**Published:** 2016-01-27

**Authors:** Elizabeth F. Daut, Glenn Lahodny, Markus J. Peterson, Renata Ivanek

**Affiliations:** 1 Schubot Exotic Bird Health Center, Department of Veterinary Pathobiology, College of Veterinary Medicine and Biomedical Sciences, Texas A&M University, College Station, Texas, United States of America; 2 Department of Mathematics, Texas A&M University, College Station, Texas, United States of America; 3 Department of Biological Sciences, University of Texas at El Paso, El Paso, Texas, United States of America; 4 Department of Veterinary Integrative Biosciences, College of Veterinary Medicine and Biomedical Sciences, Texas A&M University, College Station, Texas, United States of America; CSIRO, AUSTRALIA

## Abstract

Illegal wildlife-pet trade can threaten wildlife populations directly from overharvest, but also indirectly as a pathway for introduction of infectious diseases. This study evaluated consequences of a hypothetical introduction of Newcastle disease (ND) into a wild population of Peru’s most trafficked psittacine, the white-winged parakeet (*Brotogeris versicolurus*), through release of infected confiscated individuals. We developed two mathematical models that describe ND transmission and the influence of illegal harvest in a homogeneous (model 1) and age-structured population of parakeets (model 2). Infection transmission dynamics and harvest were consistent for all individuals in model 1, which rendered it mathematically more tractable compared to the more complex, age-structured model 2 that separated the host population into juveniles and adults. We evaluated the interaction of ND transmission and harvest through changes in the basic reproduction number (*R*_*0*_) and short-term host population dynamics. Our findings demonstrated that ND introduction would likely provoke considerable disease-related mortality, up to 24% population decline in two years, but high harvest rates would dampen the magnitude of the outbreak. Model 2 produced moderate differences in disease dynamics compared to model 1 (*R*_*0*_ = 3.63 and 2.66, respectively), but highlighted the importance of adult disease dynamics in diminishing the epidemic potential. Therefore, we suggest that future studies should use a more realistic, age-structured model. Finally, for the presumptive risk that illegal trade of white-winged parakeets could introduce ND into wild populations, our results suggest that while high harvest rates may have a protective effect on the population by reducing virus transmission, the combined effects of high harvest and disease-induced mortality may threaten population survival. These results capture the complexity and consequences of the interaction between ND transmission and harvest in a wild parrot population and highlight the importance of preventing illegal trade.

## Introduction

Illegal wildlife trade and infectious diseases are recognized conservation threats affecting wildlife populations [1–3]. Illegal and poorly regulated wildlife trade can result in overharvest and threaten population and species survival [4–7]. Introduced infectious diseases have been linked to major declines of wildlife populations [8–10], and even species extinctions [11,12]. The influence of *legal* harvest or culling on disease dynamics in wildlife populations has been examined [13–15], but the influence of *illegal* wildlife trade on the introduction and spread of infectious diseases has rarely been investigated (but for related topics see: [16–18]). The risks of introducing infectious diseases are particularly high in developing nations where illegal wildlife trade for domestic consumers flourishes and law enforcement and disease surveillance are often lacking [19–22].

Illegal trade in wild-caught birds as pets is common in many regions of the world including southeast Asia [23,24], Africa [25–28], and Latin America [29–35]. Over-exploitation for the pet trade and hunting threatens almost 40% of at-risk birds (i.e., avian species with a conservation status other than least-concern on International Union for Conservation of Nature and Natural Resources (IUCN) Red List) [36]. While songbirds are often illegally harvested in large numbers, particularly for singing competitions [37–39], parrots have historically been and continue to be the most exploited avian family for pets [36,40,41]. As a result of overharvest and other conservation threats such as destruction of suitable habitat [36,42], psittacidae is one of most the threatened bird families in the world, with 152 of the 350 extant species (43%) listed in a threatened category on the IUCN Red List [43,44]. In the Neotropics, Clarke & de By (2013), suggested that poaching is the greatest threat contributing to parrot decline [45].

Peru has among the highest parrot diversities in the world (n = 53 species) [46] and a long history of parrot harvest for the pet trade [47–49]. In a recent market study, 65% of the country’s parrot species were observed illegally for sale for domestic consumers [29]. As a case study, we used simulation modeling to evaluate a hypothetical introduction and outbreak of Newcastle disease (ND) in a population of Peru’s most trafficked psittacine, the white-winged parakeet (*Brotogeris versicolurus*) [48,50]. Threat of infectious disease and illegal harvest of wildlife should be of concern for Peru because it is a megadiverse country and a high priority area for biodiversity conservation [51].

The white-winged parakeet is a small, non-threatened, highly gregarious species that is common throughout most of its Amazonian range [52,53]. Peruvian authorities consider domestic trade of the white-winged parakeet and other native birds illegal because harvest and commercialization are conducted without proper licenses and authorizations [29,54]. During a five-year market survey in Peru, supply of white-winged parakeets was surprisingly constant throughout the year, which may stem from dual harvest techniques—taking nestlings from easily accessible nests located in arboreal termite mounds during the breeding season, and capturing adult parakeets throughout the year using mist nets at roost and feeding sites [49]. It is difficult to determine whether harvest is sustainable [55], but current harvest rates do not appear to negatively influence abundance [53].

Newcastle disease is a highly infectious and fatal viral disease caused by avian paramyxovirus serotype-1 that affects many avian species including parrots and poultry [56]. Large epidemics have occurred in poultry operations [57,58], racing pigeons [59], and free-ranging double-crested cormorants [60]. Even though vaccination programs have largely prevented recent outbreaks in commercial flocks, ND is still a serious problem in backyard poultry in rural areas throughout the developing world and in the pet trade [61–63]. In 2004, ND virus was isolated in a shipment of imported parrots and other avian species from Pakistan to Italy [64].

The ND virus spreads horizontally between healthy and infected birds through direct contact with bodily secretions from infected birds [65]. Crowded confinement typical of poultry houses or large breeding rookeries provides ideal conditions for virus transmission [65]. Disease in parrots is suspected to result from contact with infected poultry, particularly at animal markets [66,67]. Wild-caught parrots smuggled into the United States in the 1970s were suspected to have acquired ND while at animal markets in South America [58,67,68]. Subsequent outbreaks of ND in chickens cost the U.S. poultry industry millions of dollars [68]. The most common clinical signs in captive psittacine species were respiratory, but ranged from lethargy to limb paralysis [69–72]. Mortality can reach as high as 100% [68], but typically ranged from 20 to 80% [69,70,73].

White-winged parakeets are susceptible to ND [67,74]. During an outbreak in Austria, 53% (n = 32) of parakeets died from ND following importation [73]. In the early 1980s, ND was diagnosed in importation lots of white-winged parakeets from Argentina and Bolivia four times according to United States Department of Agriculture (USDA) quarantine records [75]. Newcastle disease is considered endemic in Peru [76,77]. Almost 100 outbreaks have been reported to the World Organization for Animal Health (OIE) during 2008–2014 typically in unvaccinated backyard (*criollo*) chickens and fighting cocks (e.g., [78]), with 34 outbreaks in 2014 alone [76,79]. In animal markets throughout Peru it is common to observe wild-caught parrots alongside *criollo* chickens (E. Daut, personal observation; see S3 for photographs of animal markets), thus providing the opportunity for cross-species ND transmission. Authorities frequently confiscate white-winged parakeets and immediately release individuals into the wild [29,80], typically without health evaluation because they do not have financial or diagnostic means to conduct medical screening [20,81–85]. Although ND has not been identified in the limited studies of wild psittacines to date [86–90], we expect that illegal trade provides a mechanism for ND to reach susceptible populations of white-winged parakeets due to the release of confiscated individuals infected at animal markets.

Infectious disease mathematical modeling is a useful tool for conservationists and epidemiologists to evaluate potential synergistic effects of illegal trade and disease on wildlife populations and to compare mitigating strategies [91,92]. The influence of illegal trade—specifically harvest—on pathogen transmission can be evaluated by comparing the pathogen’s basic reproduction number (*R*_*0*_) at different harvest rates [93]. *R*_*0*_ is the average expected number of secondary infections produced by one typical infectious individual introduced into a fully susceptible host population [93], and is often used as a threshold value to determine whether or not a pathogen can invade and persist in the population (*R*_*0*_ ≥ 1) or fades out (*R*_*0*_ < 1).

Under certain density-dependent pathogen transmission conditions, harvest can decrease transmission and is the reason culling can be effective to prevent disease spread [94,95]. In other cases, where birth rate in a host population is under strong density-dependence so that harvest would stimulate natality, harvest can increase the number of susceptible individuals in the population and subsequent disease prevalence and disease-induced mortality [13]. Because both harvest and diseases can be age selective, age structure can be an important demographic component in mathematical models. Recent studies have demonstrated that incorporating age structure into disease modeling can have strong, yet often unpredictable, influences on wildlife disease dynamics [96,97].

We hypothesized that (1) introducing ND into a susceptible population of white-winged parakeets would result in an outbreak with considerable mortality and (2) increasing harvest would lower disease transmission and the magnitude of the outbreak. To evaluate these hypotheses, we developed two continuous-time, mathematical models to assess whether predictions about ND dynamics would differ between a simplified model with a homogeneous bird population and a presumably more realistic, but less tractable, model with an age-structured host population. Both models were considered under different harvest scenarios and *R*_*0*_ and disease-related mortality were assessed using a combination of analytical and numerical approaches. We conducted sensitivity and scenario analyses to evaluate the robustness of the models’ results in the presence of uncertainty to individual parameters. Lastly, we discussed the limitations and implications of our results, including the conservation relevance of illegal trade and ND emergence in native populations of white-winged parakeets.

## Materials and Methods

### Model formulation

Because ND typically results in severe but short-lived epidemics in avian species, we focused our attention on short-term infection dynamics without including density-dependent responses from the host population to the disease-induced mortality (e.g., increased fecundity). However, given the possibility of long-term chronic infections in parrots [69], we did consider an endemic state where ND persists in the population.

**Fig 1:**
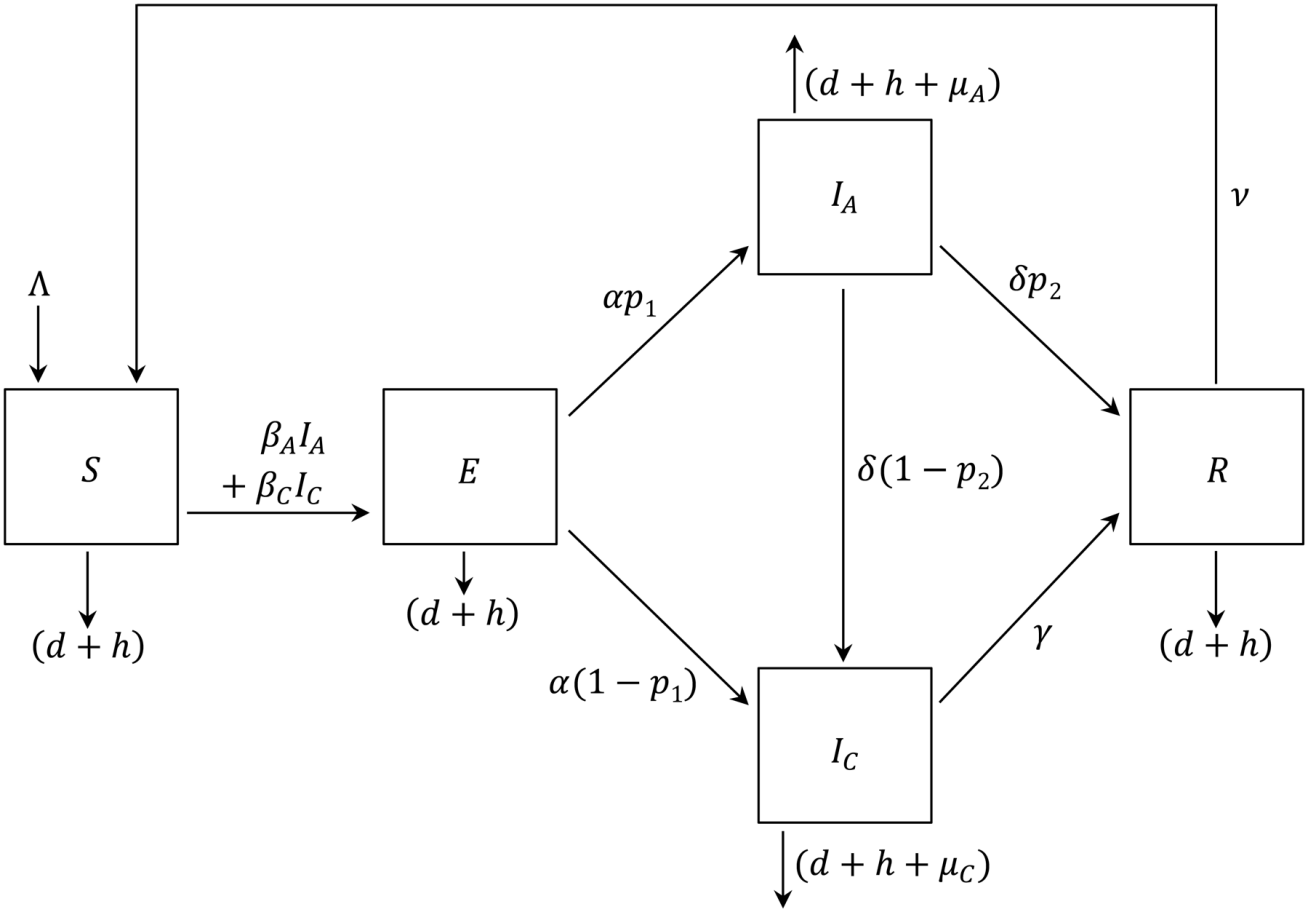
Compartmental diagram of the dynamics of Newcastle disease in a homogeneous population of white-winged parakeets (model 1). Five transition states include: susceptible (*S*), exposed (*E*), acutely-infected (*I*_*A*_), chronically-infected (*I*_*C*_) and recovered (*R*). See Table 1 for parameter descriptions.

**Fig 2:**
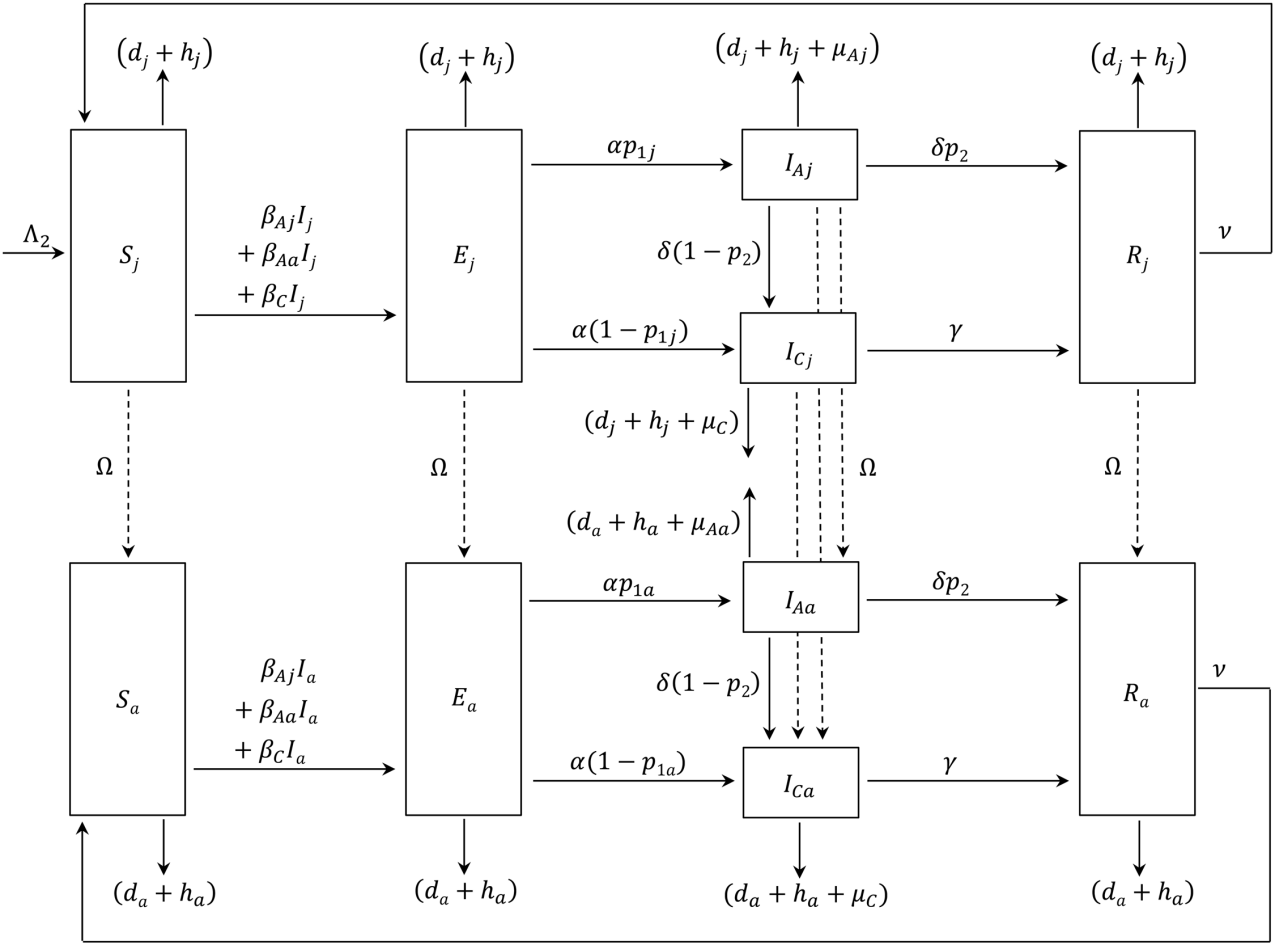
Compartmental diagram of the dynamics of the dynamics of Newcastle disease in an age-structured population of white-winged parakeets (model 2). Transition states for juvenile parakeets are: susceptible (*S*_*j*_), exposed (*E*_*j*_), acutely-infected (*I*_*Aj*_), chronically-infected (*I*_*Cj*_) and recovered (*R*_*j*_) and for adult parakeets the states are: susceptible (*S*_*a*_), exposed (*E*_*a*_), acutely-infected (*I*_*Aa*_), chronically-infected (*I*_*Ca*_) and recovered (*R*_*a*_). See Table 1 for parameter descriptions.

In model 1 (Fig 1), we described the parakeet population as homogeneous where the *SEI*_*A*_*I*_*C*_*R* model assumed no differences in the infection—transmission dynamics and harvest among age groups. The host population was divided into susceptible (*S*), exposed (*E*), acutely-infected (*I*_*A*_), chronically-infected (*I*_*C*_), and recovered (*R*) states [98]. We included two infectious stages (*I*_*A*_ and *I*_*C*_) because experimental evidence suggested two levels of severity of clinical signs, which we used as a proxy for viral shedding [69,99]. In model 2, we divided the host population into juvenile and adult stages to account for age-related differences in harvest and disease transmission and severity (Fig 2). While model 2 was demographically more realistic than model 1, it was also far more complex and less tractable, which makes its application by non-mathematicians more difficult and brings up a question of whether model 1 could be an acceptable alternative for studying interaction of infection and harvest in a wild bird population. To assure a fair comparison of results between both models, model 2 was structured to collapse into model 1 when the two age stages had the same parameter values. Both models included a baseline harvest rate (*h*_*b*_ = 1%), which we assumed was the current harvest rate of white-winged parakeets in Peru, based on the fact that this species is highly harvested but the population size remains stable [53]. Furthermore, we conducted scenario analyses to evaluate the interaction of ND dynamics and additional plausible harvest (*h1*) on *R*_*0*_ and host population size.

Our mathematical framework started with the following assumptions:

1. The host population was a single, free-mixing population and individual parakeets were equally likely to encounter an infected individual. This was a realistic assumption given the gregarious nature of white-winged parakeets and the large number of individuals at communal roost sites, up to 700 to 1,000 individuals [100,101].
2. The host population was stable under initial conditions, closed to immigration and emigration, and parakeet natality and mortality were not under the influence of density dependence. While these conditions may not always hold true, they were useful simplifying assumptions that helped isolate the evaluation of harvest and ND dynamics.
3. Both sexes were equally affected by ND virus [62].
4. At hatching, chicks were susceptible to ND virus. There is limited evidence that psittacine chicks can receive maternal antibodies through the egg [102]; however, no studies have evaluated whether protective maternal ND-antibodies are transferred to psittacine chicks.
5. Transmission of ND was density-dependent where the number of contacts per unit time was proportional to the number of individuals in the population [103]. Density-dependent transmission was a reasonable assumption and has been suggested for systems where the pathogen is transmitted through random contact among individuals and/or by aerial transmission [104,105]. Airborne transmission was considered to contribute to spread of the ND virus near poultry facilities [106,107] and in captive Neotropical psittacines [69].

We used a system of ordinary differential equations (ODEs) to describe transmission of ND in white-winged parakeets for each model (Eqs 1–5 for model 1 and Eqs 6–15 for model 2). For both models, the initial (time *t* = 0) susceptible population, *S*(0), was set equal to the total population, *N*(0), minus one individual, which represented an acutely-infectious parakeet introduced into the population, *I*_*A*_(0) = 1 and *I*_*Aa*_(0) = 1 for models 1 and 2, respectively. All remaining stages were set to zero. Model simulations and analyses were conducted in Matlab R2015a (MathWorks, USA); see https://github.com/NDCcodes/Daut_ND-matlab-codes/tree/master or S1 File for Matlab modeling codes.

### Model 1

$$\frac{dS}{dt}=\;\Lambda -\left(d+h\right)S-\beta_{A}SI_{A}-\beta_{C}SI_{C}+\nu R$$ (1)

Where Λ = *N*(*d*+*h*) and *N* changes over time as *N*(*t*) = *S*(*t*)+*E*(*t*)+*I*_*A*_(*t*)+*I*_C_(*t*)+*R*(*t*)

$$\frac{dE}{dt}\;=\;\beta_{A}SI_{A}+\beta_{C}SI_{C}-\left(d+h+\alpha \right)E$$ (2)

$$\frac{dI_{A}}{dt}=\;\alpha p_{1}E-\left(d+h+\mu_{A}+\delta \right)I_{A}$$ (3)

$$\frac{dI_{C}}{dt}=\;\alpha \left(1-p_{1}\right)E+\delta \left(1-p_{2}\right)I_{A}-\left(d+h+\mu_{C}+\gamma \right)I_{C}$$ (4)

$$\frac{dR}{dt}=\;\delta p_{2}I_{A}+\;\gamma I_{C}-\left(d+h+\nu \right)R$$ (5)

### Model 2

Juvenile:

$$\frac{dS_{j}}{dt}=\Lambda_{2}-\left(d_{j}+h_{j}+\;\Omega \right)S_{j}-\beta_{Aj}S_{j}I_{Aj}-\beta_{C}S_{j}I_{Cj}-\beta_{Aa}S_{j}I_{Aa}-\beta_{C}S_{j}I_{Ca}+\nu R_{j}$$ (6)

Where $\Lambda_{2}=\frac{N\left(d_{j}+h_{j}+\Omega \right)\left(d_{a}+h_{a}\right)}{d_{a}+h_{a}+\Omega }$ and *N* changes over time as *N*(*t*) = *N*_*j*_(*t*)+*N*_*a*_(*t*) where *N*_*j*_(*t*) = *S*_*j*_(*t*)+*E*_*j*_(*t*)+*I*_*Aj*_(*t*)+*I*_*Cj*_(*t*)+*R*_*j*_(*t*) and *N*_*a*_(*t*) = *S*_*a*_(*t*)+*E*_*a*_(*t*)+*I*_*Aa*_(*t*)+*I*_*Ca*_(*t*)+*R*_*a*_(*t*)

$$\frac{dE_{j}}{dt}=\;\beta_{Aj}S_{j}I_{Aj}+\beta_{C}S_{j}I_{Cj}+\beta_{Aa}S_{j}I_{Aa}+\beta_{C}S_{j}I_{Ca}-\left(d_{j}+h_{j}+\Omega +\alpha \right)E_{j}$$ (7)

$$\frac{dI_{Aj}}{dt}\;=\;\alpha p_{1j}E_{j}-\left(d_{j}+h_{j}+\Omega +\mu_{Aj}+\delta \right)I_{Aj}$$ (8)

$$\frac{dI_{Cj}}{dt}=\;\alpha \left(1-p_{1j}\right)E_{j}+\delta \left(1-p_{2}\right)I_{Aj}-\left(d_{j}+h_{j}+\Omega +\mu_{C}+\gamma \right)I_{Cj}$$ (9)

$$\frac{dR_{j}}{dt}=\;\delta p_{2}I_{Aj}+\gamma I_{Cj}-\left(d_{j}+h_{j}+\Omega +\nu \right)R_{j}$$ (10)

Adult:

$$\frac{dS_{a}}{dt}=\Omega S_{j}-\left(d_{a}+h_{a}\right)S_{a}-\beta_{Aa}S_{a}I_{Aa}-\beta_{C}S_{a}I_{Ca}-\beta_{Aj}S_{a}I_{Aj}-\beta_{C}S_{a}I_{Cj}+\nu R_{a}$$ (11)

$$\frac{dE_{a}}{dt}=\Omega E_{j}+\beta_{Aa}S_{a}I_{Aa}+\beta_{C}S_{a}I_{Ca}+\beta_{Aj}S_{a}I_{Aj}+\beta_{C}S_{a}I_{Cj}-\left(d_{a}+h_{a}+\alpha \right)E_{a}$$ (12)

$$\frac{dI_{Aa}}{dt}=\Omega I_{Aj}+\alpha p_{1a}E_{a}-\left(d_{a}+h_{a}+\mu_{Aa}+\delta \right)I_{Aa}$$ (13)

$$\frac{dI_{Ca}}{dt}=\Omega I_{Cj}+\alpha \left(1-p_{1a}\right)E_{a}+\delta \left(1-p_{2}\right)I_{Aa}-\left(d_{a}+h_{a}+\mu_{C}+\gamma \right)I_{Ca}$$ (14)

$$\frac{dR_{a}}{dt}=\Omega R_{j}+\delta p_{2}I_{Aa}+\gamma I_{Ca}-\left(d_{a}+h_{a}+\nu \right)R_{a}$$ (15)

### Basic reproduction number (*R*_*0*_)

To calculate *R*_*0*_ for our systems of ODEs, we used the next-generation method (NGM) [108]. See S2 file for the full derivations of *R*_*0*_. For model 1 the derived expression for *R*_*0*_ (Eq A1) was:

$$R_{0}=R_{0A}+R_{0C}.$$

The terms *R*_*0A*_ and *R*_*0C*_ represent the average number of secondary infections resulting from interactions between susceptible and acutely- and chronically-infected hosts, respectively. For model 2 the derived expression for *R*_*0*_ (Eq A2) was:

$$R_{0}=\frac{1}{2}\left[R_{01}+R_{02}+\sqrt{{\left(R_{01}-R_{02}\right)}^{2}+4R_{03}R_{04}}\right].$$

The terms *R*_*01*_ and *R*_*02*_ represent the average number of secondary juvenile or adult infections, respectively, produced by introduction of one exposed juvenile *E*_*j*_ during its entire infectious period. The terms *R*_*03*_ and *R*_*04*_ represent the average number of secondary juvenile or adult infections produced by introduction of one exposed adult *E*_*a*_ during its entire infectious period, respectively.

### Parameter estimates

Parameter notations, definitions, mean values, distributions for sensitivity analyses, and sources of information are described below and in Table 1. All demographic parameters except population size were constant and disease parameters were allowed to vary around their corresponding mean (baseline) values.

**Table 1:** Definitions and values of parameters for the model of Newcastle disease (ND) transmission in a homogeneous (model 1) and age-structured (model 2) population of wild white-winged parakeets.

Notation	Definition (unit)	Baseline value	Sensitivity analysis	5^th^ and 95^th^ percentiles	Model	Source
*Population Dynamics Parameters*						
*N*(0)	Initial number of individuals of white-winged parakeets in a typical flock in Ucayali, Peru	200	Log-normal	109, 329	1	[100,109]
			(ln(189), ln(1.4))			
*N*_j_,	Initial number of individual juvenile (*N*_*j*_) and adult (*N*_*a*_) white-	*N*_*j*_ = 0.08*N*,	-	-	2	Estimated for *D*_*d*_ = 5 years^a^
		*N*_*a*_ = 0.92*N*				
*N*_*a*_	winged parakeets in a typical post-breeding flock in Ucayali, Peru					
*D*_*d*_	Life expectancy (year)	5.0^b^	-	-	1, 2	[110]
*d*	Natural mortality rate (day^-1^)	1/*D*_*d*_	-	-	1, 2	-
*D*_*Ω*_	Duration of juvenile stage (day)	135	-	-	2	Informed from: [102,111–116]
*Ω*	Rate of leaving juvenile stage (day^-1^)	1/*D*_*Ω*_	-	-	2	-
*d*_*j*_	Natural juvenile mortality rate (day^-1^)	1/–*D*_*Ω*_/ln(0.6)^c^	-	-	2	[115,117]
*d*_*a*_	Natural adult mortality rate (day^-1^)	1/(1/*d*–1/*d*_*j*_)	-	-	2	-
*h*_*b*_	Current (baseline) harvest rate (year^-1^)^d^	1%	-	-	1, 2	[53]
*h1*	Additional harvest rate (year^-1^)^d^	0^e^	-	-	1, 2	-
*h*	Total harvest rate (year^-1^)^d^	*h*_*b*_+*h1*	-	-	1, 2	-
*h*_*j*_	Total juvenile harvest rate (year^-1^)^d^	0.4*h*	-	-	2	Informed from: [118]
*h*_*a*_	Total adult harvest rate (year^-1^)^d^	0.6*h*	-	-	2	Informed from: [118]
*Infection Parameters*						
*β*_*A*_	Transmission rate from an acutely-infected parakeet (individual^-1^ day^-1^)	0.00107	Uniform (0.00107*0.5, 0.00107*1.5)	0.000601, 0.0016	1, 2	[119]
*I*_*r*_	Infectiousness reduction coefficient for chronically-infected parakeets	0.1	Uniform (0.05, 0.15)	0.055, 0.145	1, 2	Assumed
*β*_*C*_	Transmission rate from a chronically-infected parakeet (individual^-1^ day^-1^)	*β*_*A*_*I*_*r*_	-	-	1, 2	-
*c*_*βA*_	Age-dependent acute transmission factor	0.25	Uniform (0.25*0.5, 0.25*1.5)	0.1375, 0.3625	2	Informed from: [120–122]
*β*_*Aj*_	Transmission rate for acutely-infected juvenile parakeet (individual^-1^ day^-1^)	(1+*c*_*βA*_*)β*_*A*_	-	-	2	-
*β*_*Aa*_	Transmission rate for acutely-infected adult parakeet (individual^-1^ day^-1^)	(1–*c*_*βA*_*)β*_*A*_	-	-	2	-
*D*_*α*_	Duration of infected but not yet infectious stage (day)	5.5	Exponential	0.3, 16.5	1, 2	[56]
			(1/5.5)			
*α*	Rate of becoming infectious (day^-1^)	1/*D*_*α*_	-	-	1, 2	-
*D*_*δ*_	Duration of acute-infectious stage (day)	30	Exponential	1.5, 89.9	1, 2	[99]
			(1/30)			
*δ*	Rate of leaving acute-infectious stage (day^-1^)	1/*D*_*δ*_			1, 2	-
*D*_*γ*_	Duration of chronic-infectious stage (day)	39	Exponential	2.0, 116.8	1, 2	[69]
			(1/39)			
*γ*	Rate of leaving chronic-infectious stage (day^-1^)	1/*D*_*γ*_	-	-	1, 2	-
*D*_*ν*_	Duration of immunity (day)	243	Uniform	132.25, 352.75	1, 2	[69]
			(120, 365)			
*ν*	Rate of losing immunity (day^-1^)	1/*D*_*ν*_	-	-	1, 2	-
*p*_*1*_	Probability of acute infection	0.625	Uniform	0.5125, 0.7375	1	[69]
			(0.5, 0.75)			
*p*_*2*_	Probability of recovery from acute infection	0.5	Uniform	0.3875, 0.6125	1, 2	Informed from: [123–125]
			(0.375, 0.625)			
*p*_*1j*_	Probability of acute juvenile infection	0.75	Uniform	0.6375, 0.8625	2	Informed from: [65,68,126,127]
			(0.625, 0.875)			
*p*_*1a*_	Probability of acute adult infection	0.5	Uniform	0.3875, 0.6125	2	Informed from: [65,68,126,127]
			(0.375, 0.625)			
*pd*_*A*_	Probability of acute disease-related death	0.25	Uniform	0.115, 0.385	1, 2	Informed from: [73]
			(0.1,0.4)			
*μ*_*A*_	Disease-related mortality rate for acutely-infected parakeet (day^-1^)	*pd*_*A*_/*D*_*δ*_	-	-	1, 2	-
*pd*_*C*_	Probability of chronic disease-related death	0.075	Uniform	0.03, 0.12	1, 2	[69,99]
			(0.025, 0.125)			
*μ*_*C*_	Disease-related mortality rate for chronically-infected parakeet (day^-1^)	*pd*_*C*_*/D*_*ν*_	-	-	1, 2	-
*c*_*m*_	Age-dependent acute mortality factor	0.25	Uniform	0.1375, 0.3625	2	Informed from: [65,68,126,127]
			(0.25*0.5, 0.25*1.5)			
*μ*_*Aj*_	Disease-related mortality rate for acutely-infected juvenile parakeet (day^-1^)	(1+*c*_*m*_)*μ*_*A*_	-	-	2	-
*μ*_*Aa*_	Disease-related mortality rate for acutely-infected adult parakeet (day^-1^)	(1–*c*_*m*_)*μ*_*A*_	-	-	2	-

^a^ Proportions of *N*_*j*_ and *N*_*a*_ were fixed according to disease-free equilibrium (DFE) conditions and remained constant for all iterations. When life expectancy *D*_*d*_ = 2, *N*_*j*_ = 0.22*N*, *N*_*a*_ = 0.78*N*; and when *D*_*d*_ = 9, *N*_*j*_ = 0.05*N*, *N*_*a*_ = 0.95*N*.

^b^ Scenario analysis was conducted at 2, 5, and 9 years.

^c^ 0.6 represents 40% mortality in the juvenile stage.

^d^ Annual rates were prorated to daily rates.

^e^ Scenario analysis for *hl* was conducted at 0, 2, 5, and 10%.

### Model 1

#### Host demography and harvest

We defined the initial population of parakeets as (*N*(0) = 200), a commonly reported flock size [100,109], which included those birds roosting together at a communal site throughout the year, except during the breeding season when pairs separate for nesting. We set population recruitment (*Ʌ*) equal to the sum of natural mortality (*d*) and the current (baseline) harvest (*h*_*b*_) to maintain a stable population in absence of additional harvest (*hl*), so that *Ʌ* = *N*(*d*+*h*_*b*_) [53]. Daily natural mortality was calculated as the inverse of the mean life expectancy (*D*_*d*_ = 5 years) from captive white-winged parakeets, so that *d* = 1/*D*_*d*_ [110]. Baseline harvest of white-winged parakeets was set at a conservatively low constant daily rate for a cumulative total annual harvest of 1% (*h*_*b*_ = 0.01/365).

#### Transmission rate

Transmission coefficients (*β*) for ND in psittacines have not been published; therefore, we adapted a transmission probability published for backyard poultry that was considered to reasonably represent ND dynamics in wild white-winged parakeets. In a mathematical model describing density-dependent ND dynamics in a backyard chicken flock, Johnston [119] estimated 3% infection probability during a 14-day period. We prorated this probability to a per day rate for our population size and used it as a baseline transmission rate for acutely-infectious individuals, so that *β*_*A*_ = (3/14)200 individual^-1^ day^-1^ (Table 1). Chronic ND transmission rate was assumed to be a fraction (*I*_*r*_ = 10%) of the acute transmission rate, so that *β*_*C*_ = *β*_*A*_*I*_*r*_.

#### Infection stages

According to the standard assumption of exponentially-distributed periods of infection [98], the reciprocal values of the mean durations in days that a parakeet spends in the exposed (*E*), acutely-infected (*I*_*A*_), chronically-infected (*I*_*C*_), and recovered (*R*) stages were used as the daily rates *α*, *δ*, *γ*, and *ν*, respectively (Table 1; Fig 1). The daily rate of becoming infectious, therefore, was the inverse of the duration of an average latent period reported for most studied avian species (*α* = 1/5.5) [56]. We based the duration of the acute-infectious stage on the study by Erickson et al. [99] where individual Neotropical psittacines (n = 48) shed ND virus on average for 30 days so that *δ* = 1/30. Although some parrots continued to chronically shed for over a year, on average individuals had stopped shedding by 39 days post infection [69], which represented our baseline recovery rate from chronic infection *γ* = 1/39 and was consistent with shedding duration observed in other avian species [122,128,129]. We used this same duration for individuals that became chronically infected directly following exposure and for those individuals transitioning from an acute infection (Fig 1). We based the immunity-loss rate on the average duration of effective ND antibody titers (> 1.2 log10) [121] in experimental infections so that the average daily rate of immunity loss was *ν* = 1/243 [69].

#### Acute and chronic infections

According to ND infection rates in psittacines in captive and experimental conditions, we assumed that all effectively contacted white-winged parakeets would become either acutely or chronically infected, with acutely-infected parakeets shedding more ND virus [69,70,72,130]. Following Erickson et al.’s [69] experimental study, we considered that 63% (*p*_*1*_) of newly infected white-winged parakeets would become acutely infected, and 1–*p*_*1*_ would become chronically infected (Fig 1). After acute infection, parakeets could recover or become chronically infected where individuals continued to shed virus but at lower levels. Little has been published regarding recovery or progression to a chronic ND state; however, a chronic-like state is common following live ND vaccination and has been considered a concern for environmental contamination and infection spread [123–125]. Therefore, we considered that 50% of acutely-infected individuals would recover (*p*_*2*_), while the remainder (1–*p*_*2*_) would become chronically infected.

#### Disease-related mortality

Mortality from ND was > 50% in captive white-winged parakeets [73]. Under free-ranging conditions, we assumed that mortality would be lower and considered that 25% of individuals would die from ND during the acute-infectious period (30 days) making our daily rate, *μ*_*A*_ = 0.25/30 (Table 1). To obtain disease-related mortality in chronically-infected parakeets, we averaged mortality recorded in experimentally-infected psittacines during the nine days following the acute-infection period so that *μ*_*C*_ = 0.075/39 [69,99].

### Model 2

#### Host demography and harvest

We set the initial population size of the two age classes according to their distribution at the disease-free equilibrium with life expectancy (*D*_*d*_), so that *N*(0) = *N*_*j*_ + *N*_*a*_. For instance, when *D*_*d*_ = 5 years and *N*(0) = 200, *N*_*j*_ = 16 (8%) and *N*_*a*_ = 184 (92%) (Table 1). We estimated the duration of the juvenile stage based on two factors: (1) the age when juveniles develop immune-competence, and (2) when their survival rate starts to increase. Development of immuno-competence in psittacines and other altricial species is poorly understood [131,132], but appears to occur between six weeks [102,111] and five months [112]. Considering juvenile survival, it is widely recognized that juveniles of many avian species suffer their highest mortality during the first several months after fledging [113–116], which for white-winged parakeets occurs at roughly six weeks of age [109]. Combining these two factors we considered that juveniles transition to adults at *D*_*Ω*_ = 135 days (4.5 months), meaning that the maturation rate was *Ω* = 1/135.

We fixed juvenile mortality at 40% during the 135-day long juvenile period [115,117]. The corresponding juvenile population was modeled as an exponential decay *S*_*j*_(t) = *S*_*j*_(0)e^(–dj^*^t)^ so that 60% of the population remained after the juvenile period. The daily natural mortality was then estimated as *d*_*j*_ = 1/(–*D*_*Ω*_/ln(0.6)) = 1/264. Adult daily natural mortality *d*_*a*_ was calculated from the difference between the natural mortality already defined for model 1 (*d*) and the juvenile mortality (*d*_*j*_), so that *d*_*a*_ = 1/(1/*d*–1/*d*_*j*_) (Table 1). According to market survey data in Peru, roughly 20% more adult white-winged parakeets were harvested annually than juveniles [118], therefore, we set 60% of the total harvest (*h*) to represent adults (*h*_*a*_ = 0.6*h*) and 40% juveniles (*h*_*j*_ = 0.4*h*).

#### Transmission rate

Transmission coefficients (*β*) for ND in juvenile or adult psittacines have not been published; however, acutely-infected juvenile chickens, pigeons, and other species were considered more infectious than adults [120–122,126,133,134]. It is reasonable to assume that the same would be true for juvenile parakeets. For simplicity, we parameterized an acute-transmission factor (*c*_*βA*_) to represent a 25% increase for acutely-infected juveniles over the baseline rate of transmission (*β*_*A*_) in model 1, so that *β*_*Aj*_ = (1+*c*_*βA*_)*β*_*A*_, and a 25% decrease for adult transmission below baseline so that *β*_*Aa*_ = (1–*c*_*βA*_)*β*_*A*_ (Table 1; Fig 2). We used the same chronic transmission rate (*β*_*C*_) for juveniles and adults as in model 1. The transition rates for leaving infectious states, *α*, *δ*, *γ*, and *ν*, used in model 2 were previously defined for model 1 (Table 1).

#### Acute and chronic infections and disease-related mortality

In natural outbreaks and experimental studies, juvenile birds frequently disproportionately suffered acute clinical signs and high mortality following ND exposure compared to adults [65,68,126,127]. Therefore, we considered that 75% of juvenile white-winged parakeets would become acutely infected following exposure (*p*_*1j*_ = 0.75), while 1–*p*_*1j*_ would become chronically infected (Table 1; Fig 2). For adults, we considered a 50% probability of becoming acutely or chronically infected, *p*_*1a*_ and 1–*p*_*1a*_ = 0.5. The proportion of acutely-infected individuals that would recover (*p*_*2*_) versus becoming chronically infected was defined as in model 1. We used an acute-mortality factor (*c*_*m*_) to estimate a ± 25% age-related difference so that disease-related mortality for acutely-infected juveniles was *μ*_*Aj*_ = (1+*c*_*m*_)*μ*_*A*_ and for adults, *μ*_*Aa*_ = (1–*c*_*m*_)*μ*_*A*_. Little is known about age-related mortality from chronic ND infection. We assumed that little difference existed among juveniles and adults and, therefore, used the parameter *μ*_*C*_ from model 1 for both.

### Scenario analysis

Because life expectancy (*D*_*d*_) is unknown in the wild, we conducted a scenario analysis to compare the baseline scenario (*D*_*d*_ = 5 years) with two extreme scenarios (*D*_*d*_ = 2 and *D*_*d*_ = 9 years) for both models. Also, we evaluated the effect of additional harvest (*hl*) at 2, 5, and 10% along with the 1% annual baseline (*h*_*b*_) harvest (so that the total harvest *h* = *h*_*b*_ + *hl*) through two scenarios for each model. In the first scenario, population recruitment was fully compensated to include baseline and additional harvest to maintain a stable population in absence of infection, so that *Ʌ* = *N*(*d*+*h*) in model 1 and *Ʌ*_2_ = *N*(*d*_*j*_+*h*_*j*_+*Ω*)(*d*_*a*_+*h*_*a*_)/(*d*_*a*_+*h*_*a*_+*Ω*) in model 2 (Table 1). In the second scenario, additional harvest and infection were introduced simultaneously into the host population, but additional harvest was uncompensated by recruitment and both additional harvest and infection-induced mortality caused the population to decline so that *Ʌ*_*u*_ = *N*(*d*+*h*_*b*_) in model 1 and *Λ*_*2u*_ = *N*(*d*_*j*_+*h*_*bj*_+*Ω*)(*d*_*a*_+*h*_*ba*_)/(*d*_*a*_+*h*_*ba*_+*Ω*) in model 2, where *h*_*bj*_ = 0.4*h*_*b*_ and *h*_*ba*_ = 0.6*h*_*b*_. These two scenarios provided a means to evaluate the interaction between harvest and disease under two different host population reproductive responses to additional harvest (i.e., compensated additional harvest by increased natality in scenario 1 and uncompensated additional harvest with stable natality in scenario 2).

### Sensitivity analysis

We examined the sensitivity of the predicted disease-related population decline and *R*_*0*_ to parameters’ uncertainties (Table 1). Selected parameter distributions were supported by the literature; population size was Log-normally distributed [135], values for *D*_*α*_, *D*_*δ*_, and *D*_*γ*_ were exponentially distributed [98], and the remaining parameters with unknown distribution were uniformly distributed (Table 1). We performed a sensitivity analysis by simulating 10,000 iterations of the (1) *R*_*0*_ expressions (Eq A1 and A2) and (2) ODE models (Eqs 1–5 and 6–15) run up to 10,000 days. Parameters were varied simultaneously using the Monte Carlo method and Latin Hypercube sampling that randomly-selected parameter values from their respective distributions [136]. With this number of iterations, *R*_*0*_ was estimated with a precision of +/- 0.08.

Spearman’s correlation coefficient (*r*_*s*_) was used to calculate and test correlation between each uncertain parameter and *R*_*0*_ (Table 1). We used *α* = 0.05 as the statistical significance, which was adjusted for multiple testing by implementing the Bonferroni correction. The corrected significance level was *α* = 0.0045 for model 1 and *α* = 0.0028 for model 2. To explore how parameters interacted to create conditions of disease-free (*R*_*0*_ < 1) or endemic (*R*_*0*_ ≥ 1) states, we used the parameter values and the associated *R*_*0*_ estimates from 10,000 iterations per scenario to construct classification trees—binary decision trees that identify the most influential parameters in predicting disease-free or endemic conditions for our homogeneous and age-structured populations [137]. We combined results from all of our scenario analyses on life expectancy (*D*_*d*_) and additional harvest (*h1*), for a total of 120,000 data points (iterations) to determine whether these two, and other, model parameters were important predictors of disease-free or endemic states. Classification trees were built using the rpart package in R software [138] and we used the gini index as a measure of node impurity along with a 10-fold cross-validation to select the tree with the smallest misclassification error [139].

In a separate analysis, we compared differences in *R*_*0*_ estimates produced by models 1 and 2, by starting with equal parameters so that each model produced the same *R*_*0*_ (i.e., we set the age-structured parameters to be equal and to match those in model 1). We then varied the age-structured parameters (e.g., *c*_*βA*_, *c*_*m*_, *p*_*1j*_, and *p*_*1a*_) one at a time to evaluate their individual influence on *R*_*0*_. This provided a method to compare results between the two models and to identify which parameters made the biggest difference in *R*_*0*_ estimates.

## Results

### Dynamics of ND transmission, *R*_*0*_, and harvest

**Fig 3:**
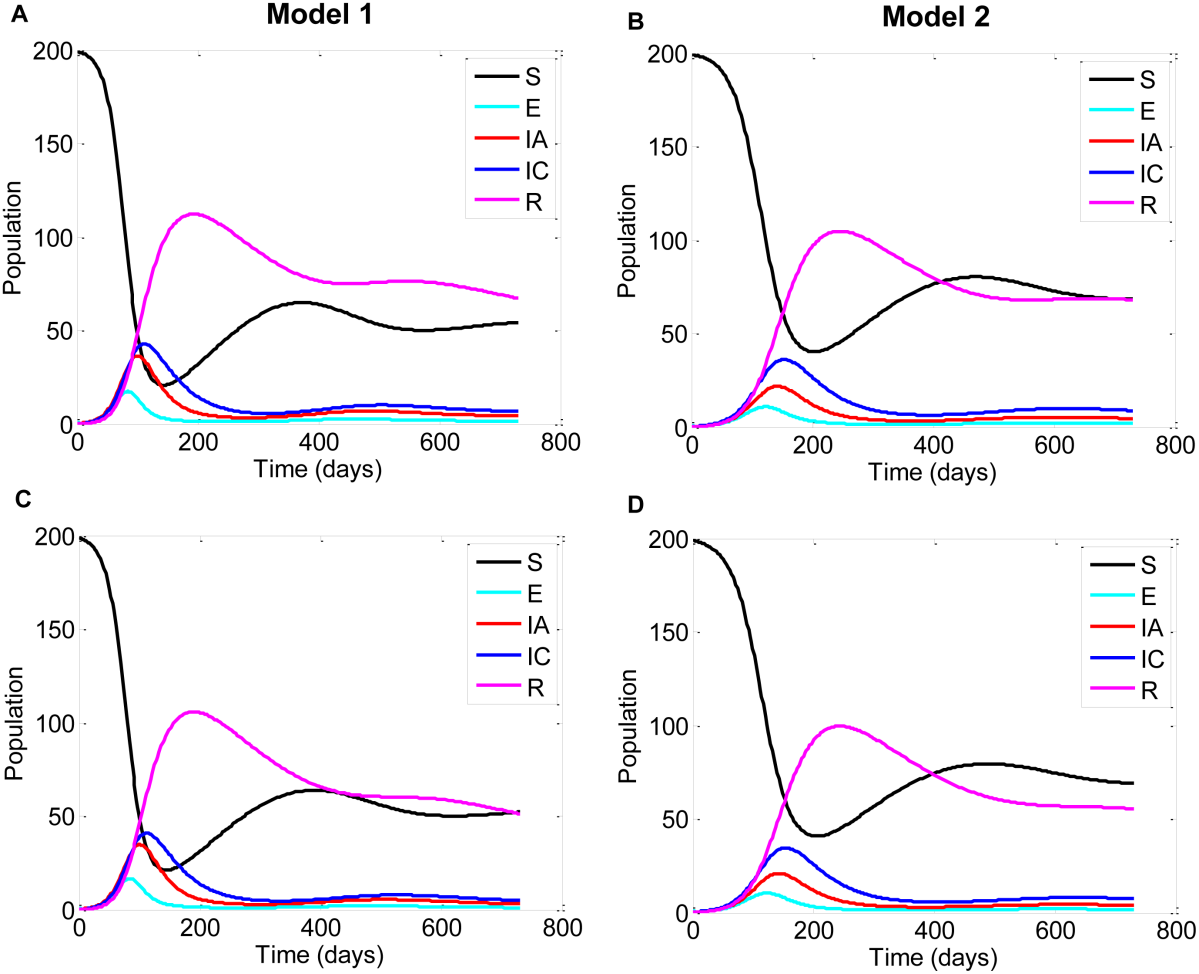
Deterministic two-year time trajectories for Newcastle disease transmission. Simulated outbreak dynamics from homogeneous (model 1) and age-structured (model 2) populations of white-winged parakeets with (A-B) no additional harvest (*hl* = 0%) and (C-D) 10% additional (uncompensated) harvest (*hl* = 10%). Here uncompensated additional harvest means that the population natality did not increase to compensate population decline due to additional harvest. Depicted states of infection are: susceptible (*S*), exposed (*E*), acutely-infected (*IA*), chronically-infected (*IC*) and recovered (*R*). Age-structured panels (B, D) show summed juvenile and adult parakeets for each infection state. See S1 Fig for separate juvenile and adult trajectories for model 2.

Our baseline *R*_*0*_ estimates without additional harvest were 3.63 and 2.66 for models 1 and 2, respectively (Table 2). These baseline values were similar to mean *R*_*0*_ estimates obtained from 10,000 simulations. The distribution range of the *R*_*0*_ estimates (represented by the 5^th^ and 95^th^ percentiles) were slightly wider in model 1 compared to model 2 (Table 2). With just baseline harvest (i.e., no additional harvest), 21 and 28% of *R*_*0*_ simulated values were < 1 for models 1 and 2, respectively, meaning that ND failed to become established in the host population (Table 2). The majority of simulated *R*_*0*_ estimates were < 5 for both models (77% and 87%, respectively). The baseline outbreak dynamics peaked at approximately 120 days for the homogeneous population of white-winged parakeets (Fig 3A), and closer to 150 days with less infected individuals and longer duration in the age-structured population (Fig 3B). Short-term ND dynamics oscillated in both models, but the second infection cycle was slightly delayed in model 2 compared to model 1(Fig 3; See S1 Fig for separate juvenile and adult trajectories).

**Table 2:** Scenario analysis of the effect of uncompensated additional harvest (*h1*) on the basic reproduction number (*R*_*0*_) and population size following introduction of Newcastle disease into a homogeneous (model 1) and age-structured (model 2) populations of white-winged parakeets. ^a^

Additional harvest, *h1* (%)	Baseline R_0_	Mean *R*_*0*_ (5^th^, 95^th^ percentiles)	Proportion of simulations where *R*_*0*_ < 1 (%)	*N*(730)^b^ (5^th^, 95^th^ percentiles)
Model 1				
0^c^	3.63	3.65 (0.35, 11.30)	21	154.8 (84.9, 254.5)
2	3.31	3.32 (0.31, 10.29)	24	149.3 (81.9, 245.0)
5	2.92	2.93 (0.28, 9.05)	27	141.4 (77.6, 231.6)
10	2.44	2.44 (0.23, 7.52)	33	129.1 (71.1, 211.2)
Model 2				
0^c^	2.66	2.62 (0.28, 7.53)	28	168.6 (94.9, 272.2)
2	2.54	2.50 (0.27, 7.18)	29	165.0 (92.9, 266.3)
5	2.39	2.34 (0.26, 6.71)	31	159.7 (89.9, 257.6)
10	2.17	2.12 (0.23, 6.07)	34	151.2 (85.2, 243.9)

^a^ For each scenario, results were based on 10,000 simulations.

^b^ Population size at day 730 post infection introduction was chosen to capture the short-term effect of harvest on the population size (the initial population size Log-normally distributed with mean of 200 and the 5^th^ and 95^th^ percentiles of 109 and 329, respectively).

^c^ In all scenarios, the uncompensated additional harvest (*h1*) was added to the 1% baseline harvest (*h*_*b*_). Here uncompensated additional harvest means that population natality did not increase to compensate the population decline due to additional harvest.

**Fig 4:**
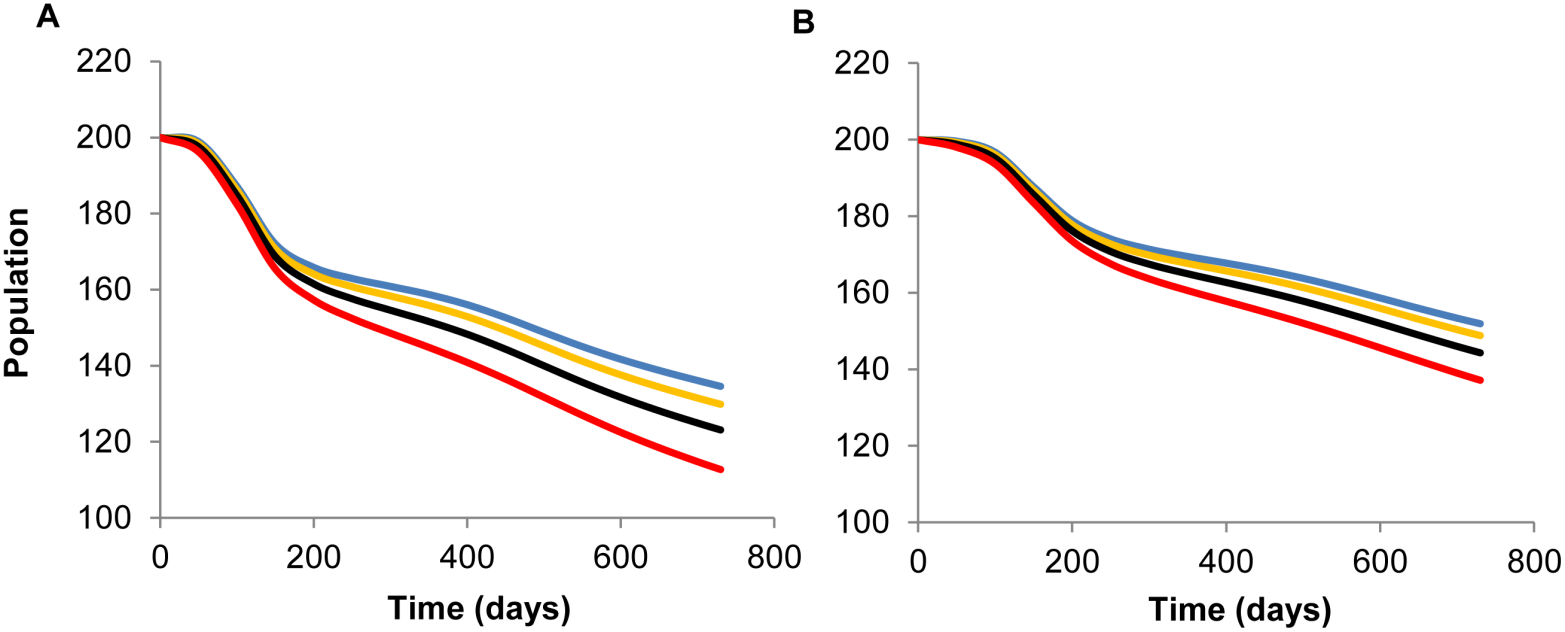
Population decline during two years post Newcastle disease introduction. Population decline in (A) homogeneous (model 1) and (B) age-structured (model 2) populations of white-winged parakeets with no additional harvest (*hl* = 0) and three additional, uncompensated harvest rates (*h1*; 0-blue, 2%-orange, 5%-black, and 10%-red). Here uncompensated additional harvest means that population natality did not increase to compensate the population decline due to additional harvest.

As shown in both panels in Fig 4, population size was relatively stable for approximately the first 20 days, after which parakeet abundance decreased sharply and similarly for all tested harvest rates until approximately 180 days in model 1 and 200 days in model 2. This sharp decline, related to the disease outbreak and disease-induced mortality shown in Fig 3, was followed by further, but slower, decline in population size. By two years (t = 730 days) post ND introduction with baseline compensated harvest, ND caused the initial population of 200 individuals to decrease by 33% in the homogeneous population (*N*(730) = 135; Fig 4A, blue line), but only by 24% (*N*(730) = 153) when age structure was considered (Fig 4B, blue line).

**Fig 5:**
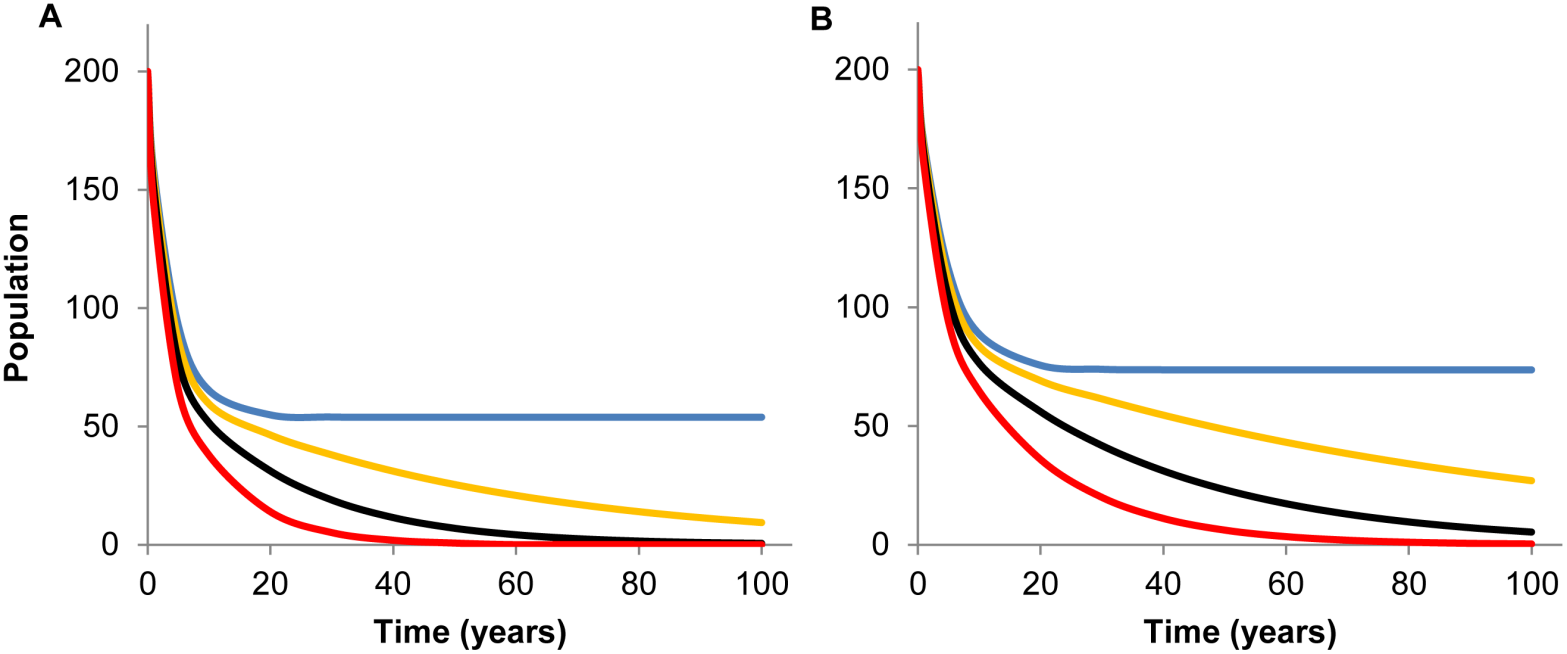
Population decline during 100 years post Newcastle disease introduction. Population decline in (A) homogeneous (model 1) and (B) age-structured (model 2) populations of white-winged parakeets with no additional harvest (*hl* = 0) and three additional uncompensated harvest rates (*h1*; 0-blue, 2%-orange, 5%-black, and 10%-red). Here uncompensated additional harvest means that population natality did not increase to compensate the population decline due to additional harvest.

By 20 years post ND introduction, homogeneous and age-structured populations decreased > 50% before stabilizing in an endemic state when no additional harvest was considered (Fig 5, blue lines). The distribution of the estimated number of parakeets from 10,000 ODE simulations was slightly wider for model 2 than model 1, which likely reflected additional uncertain parameters in model 2 (Table 2). When we fixed the initial population size to the baseline value (*N* = 200) so that we could evaluate the effects of harvest and disease-related mortality on population decline without the influence of the uncertain initial population size, the width of the 5^th^ and 95^th^ percentiles decreased by over half for both models (45–60%; S2 Fig). Uncertainly in the initial population size had much less influence on predicted *R*_*0*_ values (S3 Fig).

### Scenario analyses

**Fig 6:**
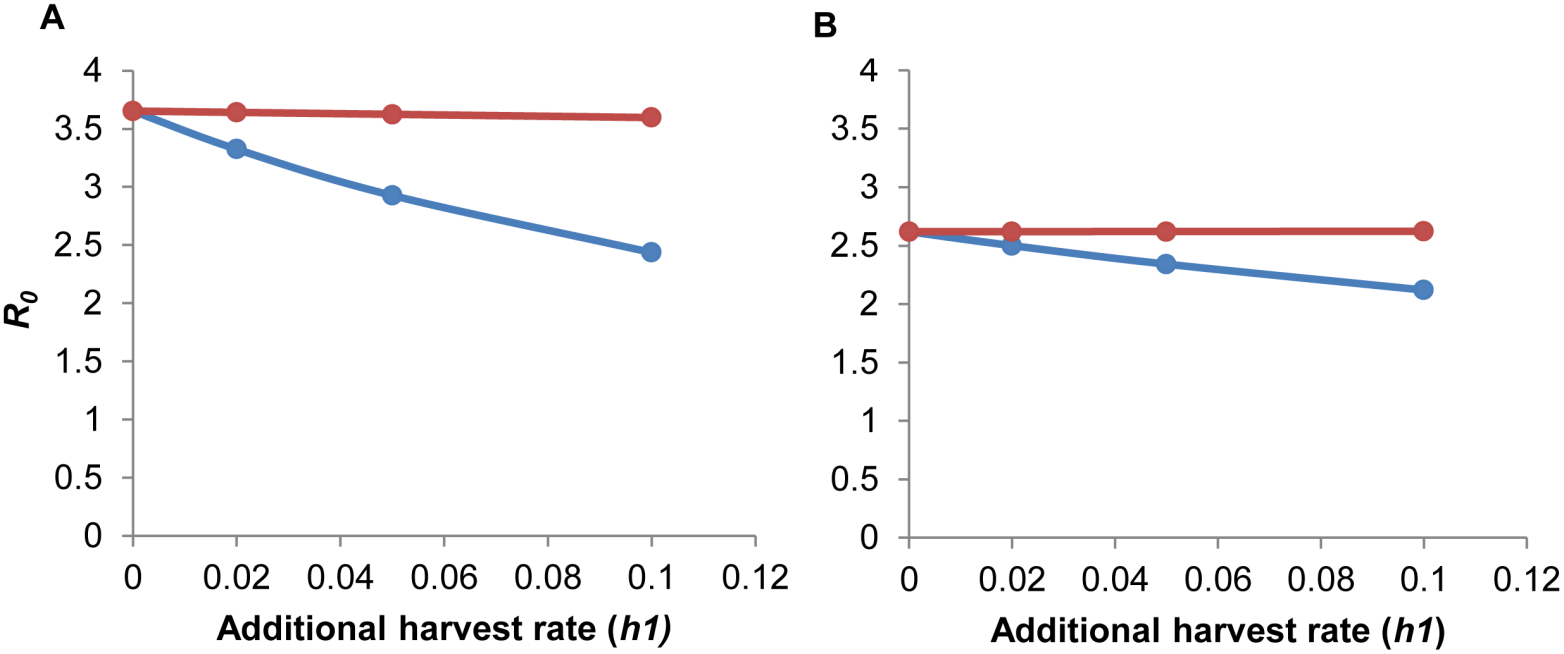
Influence of additional harvest (*hl*) on mean estimates of the basic reproduction number, *R*_*0*_. Comparison of mean *R*_*0*_ estimates under assumptions of compensated (red) and uncompensated (blue) additional harvest rates for (A) homogeneous and (B) age-structured populations of white-winged parakeets following introduction of Newcastle disease. Here compensated and uncompensated additional harvest means respectively that population natality did and did not increase to compensate the population decline due to additional harvest.

Varying life expectancy where *D*_*d*_ = 2 or 9 years produced minimal effect in ND transmission represented by mean *R*_*0*_ estimates and host population size for either homogeneous or age-structured populations of white-winged parakeets (S4 and S5 Figs). Uncompensated additional harvest dampened *R*_*0*_ in both homogeneous and age-structured populations (Table 2). Increasing the additional harvest rate from 0 to 10% increased the probability of infection fade out (Table 2). As expected, compensating for additional harvest, where natality (lambda) increased in response to higher harvest, caused *R*_*0*_ to remain stable with increasing additional harvest in both models (Fig 6); however, the confidence intervals for *R*_*0*_ estimates with compensated and uncompensated harvest largely overlapped. Overall, disease dynamics were minimally affected by higher harvest (Fig 3). The recovered class, which was the longest stage in our compartmental models, was the most affected by higher harvest rates in both models as noted by the lower number of individuals with 10% additional harvest versus baseline harvest (Fig 3, panels C–D versus panels A–B, respectively).

Higher uncompensated harvest decreased the size of both homogeneous and age-structured populations of white-winged parakeets, although the decrease was more notable in model 1 versus model 2 and the decline was steeper with higher harvest rates (Fig 4). At two years post ND introduction, 10% additional harvest decreased the homogeneous population of 200 individuals by 35%, but only by 24% in model 2 for the age-structured population (Table 2). The combination of ND and 10% uncompensated additional harvest (without density-dependent population regulation) caused the population to steadily decline, approaching zero within 35 and 60 years for models 1 and 2, respectively (Fig 5).

### Sensitivity analysis

**Fig 7:**
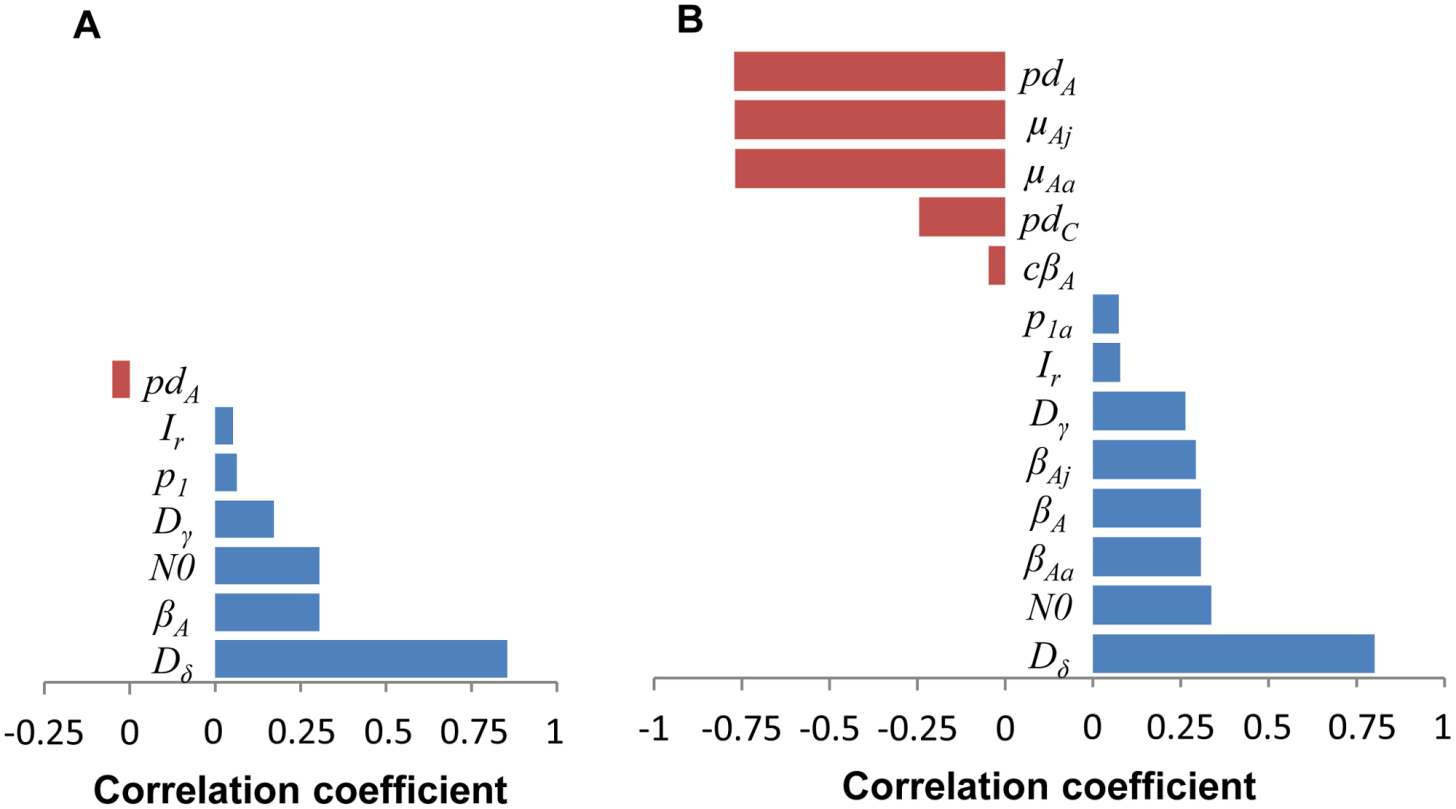
Spearman’s correlation coefficient values for models 1 and 2. Spearman’s coefficients indicating the strength of the relationship between parameters of the (A) homogeneous (model 1) and (B) age-structured (model 2) models and the basic reproduction number (*R*_*0*_) with 10% additional harvest from 10,000 simulations. Only parameters with statistically significant coefficients are shown. See Table 1 for parameter descriptions.

The duration of the acute-infectious stage (*D*_*δ*_) was the most influential (positively-correlated) parameter in determining *R*_*0*_ for both homogeneous and age-structured populations of white-winged parakeets (Fig 7). Initial population size and the transmission rate for acutely-infected individuals were also positively correlated with *R*_*0*_. The probability of acute disease-related death (*pd*_*A*_) was negatively correlated with *R*_*0*_ in both models, but had a much stronger influence in model 2 (Fig 7B). Along with *pd*_*A*_, disease-related mortalities for acutely-infected juvenile (*μ*_*Aj*_) and adult (*μ*_*Aa*_) parakeets, both of which were partially derived from *pd*_*A*_ (Table 1), were highly negatively correlated with *R*_*0*_ in model 2. The probability of chronic disease-related death (*pd*_*c*_) was likewise negatively correlated with *R*_*0*_, but only significant in model 2.

**Fig 8:**
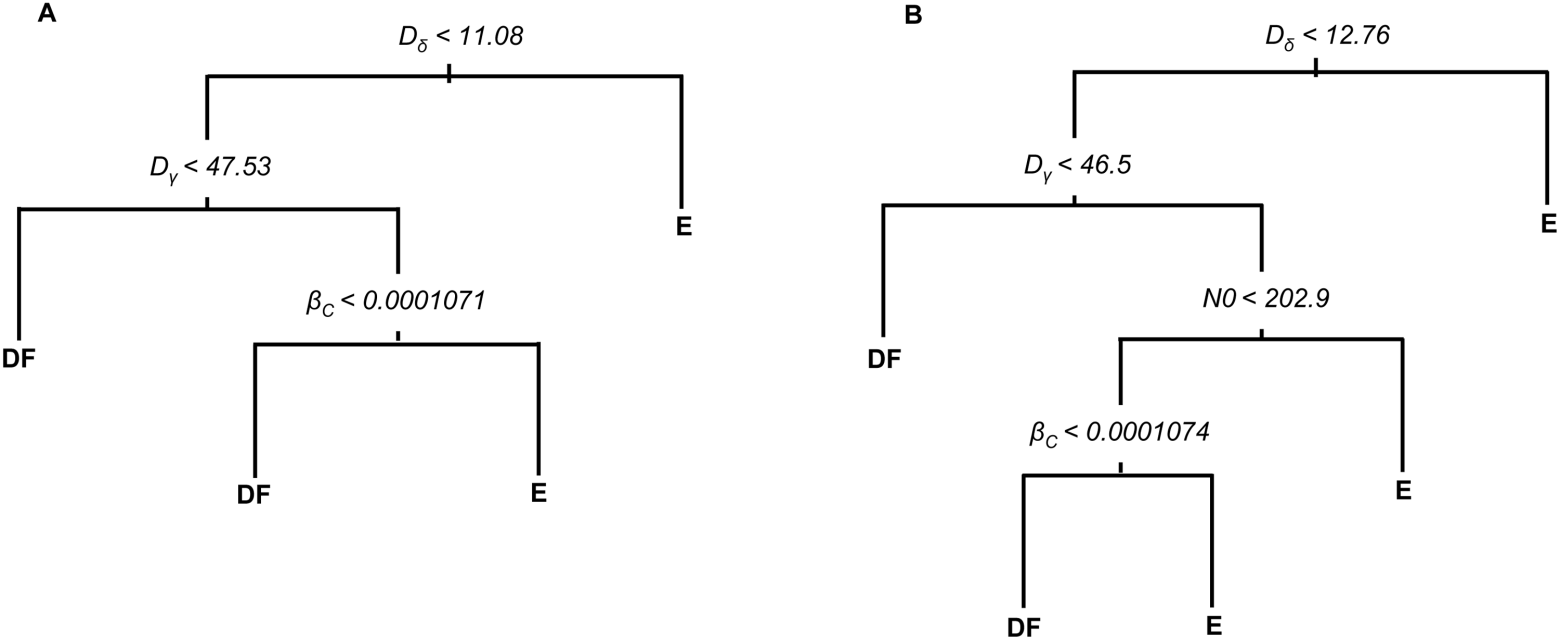
Classification trees for Newcastle disease. Classification tree for disease-free (basic reproduction number, *R*_*0*_ < 1) or endemic (*R*_*0*_ ≥ 1) conditions of Newcastle disease (ND) in (A) homogeneous and (B) age-structured populations of white-winged parakeets. The rule for data partitioning is on top of each node. For example, in panel (A), the root node rule is the duration of the acute infectious stage (*D*_*δ*_) less than 11.08 days; the subset of simulations satisfying this rule partitioned to the left daughter node and consecutively down the nodes. The terminal nodes represent disease-free (DF) or endemic (E) conditions for ND. See Table 1 for parameter descriptions.

In the correlation analyses, parameters were evaluated individually; in the multivariable classification trees analyses all uncertain parameters as well as life expectancy (*D*_*d*_) and additional harvest (*h1*) were evaluated simultaneously. Parameters *D*_*d*_ and *h1* were not identified as influential in predicting disease-free or endemic conditions. As with correlation analyses, the most optimal classification trees identified the duration of the acute-infectious stage (*D*_*δ*_) as the most important factor determining whether the infection would persist or undergo fade-out, indicated by its position closest to the root of the tree (Fig 8). By following the rule for branches to the right, endemic conditions (*R*_*0*_ ≥ 1) were predicted to occur under two scenarios in model 1: (1) when *D*_*δ*_ ≥ 11 days, and (2) when *D*_*δ*_ < 11 days, but with a duration of chronic-infectious stage *D*_*γ*_ ≥ 48 days, and a transmission rate for chronically-infected parakeets *β*_*c*_ ≥ 0.0001071 (Fig 8A). In model 2, the most optimal classification tree indicated that an endemic state was predicted by four parameters following three different pathways (Fig 8B). As with model 1, *D*_*δ*_, *D*_*γ*_, and *β*_*c*_ were influential parameters in predicting endemic ND, along with the initial population size, *N*(0). Cross-validation indicated that the predictive error rates were low and similar for models 1 and 2 (12.5% and 12.7%, respectively).

By directly comparing models 1 and 2 and the age-related parameters that differed between the two, we determined that the age-dependent acute transmission factor (*c*_*βA*_), which increased or decreased the transmission rate by 25% for acutely-infected juveniles and adults, respectively, made the biggest difference in *R*_*0*_ between the two models. Specifically, lowering the transmission rate for acutely-infected adult parakeets (*β*_*Aa*_) decreased *R*_*0*_ by 21% from 3.63 in model 1 to 2.88 in model 2. Lowering the probability of acute adult infection (*p*_*1a*_) in model 2 compared to the homogenous value (*p*_*1*_) in model 1 decreased *R*_*0*_ by 15%. The acute mortality factor (*c*_*m*_), which lowered the disease-related mortality rate for acutely-infected adult parakeets (*μ*_*Aa*_), resulted in increasing *R*_*0*_ slightly by 4% compared to model 1. The corresponding increase in juvenile parameter values associated with *c*_*βA*_, *p*_*1j*_ and *c*_*m*_, in model 2 minimally influenced *R*_*0*_ compared to model 1.

## Discussion

Our results demonstrate that illegal harvest could play an important role in virus transmission during a ND outbreak. We developed two mathematical models to compare the influence of harvest on ND dynamics in a homogeneous and an arguably more realistic age-structured population of white-winged parakeets. We determined that introduction of ND would likely provoke considerable disease-related mortality, but the magnitude of the outbreak would be dampened by high harvest rates. Incorporating age structure into the model produced moderate differences in both *R*_*0*_ and disease dynamics, primarily due to lower adult disease transmission, compared to our homogeneous population model. Most importantly, the homogenous model likely overestimated the severity of an ND outbreak and highlighted the importance of incorporating even simplistic age structure in disease modeling despite increased complexity and reduced tractability.

### *R*_*0*_ and ND outbreak

Our compartmental models demonstrated that introducing just one ND-infected individual would provoke an outbreak (*R*_*0*_ ≥ 1) in susceptible populations of white-winged parakeets with roughly 75% probability (Table 2). In the short term, without population compensation through density-dependent recruitment, population size decreased by 24–44% depending on the harvest rate and host population structure (Fig 4). Even the low end of this range, observed in the age-structured population (model 2) with baseline harvest, is a conservation concern (Fig 4B, blue line). The fact that some psittacine species can chronically shed the ND virus for extended periods [69], implies this disease has the potential to become endemic and remain in the population causing low-level disease-related mortality with significant population decline (Fig 5). In a case of another infectious disease affecting wild avian populations, Hochachka & Dhondt [140] found that epizootic *Mycoplasma* sp. conjunctivitis caused significant population decline in house finches along the eastern United States.

Our results indicate that, in the long term, ND alone would not cause population extinction; however, the combination of ND and consistently high annual harvest (10%) would provoke > 75% population decline in 20 years without a density-dependent response in fecundity (Fig 5). While we did not investigate the interaction between density-dependent effects and illegal harvest on the host population, it would be reasonable to expect that density-dependent regulation would help replenish the population by increasing reproduction [141]. Higher recruitment could potentially increase the supply of ND susceptible parakeets, and thereby exacerbate and/or prolong the outbreak [13,142]. The combination of illegal harvest and ND-related mortality could have potential devastating population-level effects, but without better demographic data for the white-winged parakeet, it is unclear from what level of decline the population could recover, or possibly stabilize at a new carrying capacity [140,143].

### Age structure

The importance of including realistic population demographic factors in disease modeling is well established [144]. In the case of ND, age structure is an important factor to consider because the disease disproportionately affects juveniles. Our modeling results demonstrated that without including age structure (model 1), the severity of a potential ND outbreak was up to 28% higher based on mean *R*_*0*_ estimates (Table 2) and disease-related population decline was 9–13% higher, depending on the harvest rate, in model 1 versus model 2 at two years post ND introduction (Fig 4). To put this in perspective, a higher *R*_*0*_ observed in model 1 compared to model 2 suggests that a higher effort is needed to control the infection, which may lead to suboptimal control strategies [145,146].

The dynamics of an ND outbreak were similar in homogeneous and age-structured populations of white-winged parakeets (Fig 3); however, the mildly prolonged infectious periods (*I*_*A*_, *I*_*C*_) in model 2 (Fig 3B–3D) would likely provide more opportunities for contact and cross-species exposure. Psittacine species, especially species of similar size, often interact at fruiting trees or clay licks [147,148]. Up to 17 species and hundreds of individuals have been identified at clay licks in Peru [149], which would provide favorable conditions for ND transmission and spread among species [150]. Expanding our age-structured model to include spatial connectivity for meta-population and interspecies interactions would provide valuable insight regarding ND dynamics on a larger scale [151,152] and should be a focus of future research.

Comparison of models 1 and 2 demonstrated that the lower *R*_*0*_ estimates predicted from model 2 primarily reflect disease dynamics in adult parakeets, specifically a lower transmission rate for acutely-infected adults (determined by *c*_*βA*_) and, to a lesser degree, a lower probability of acute adult infection. Juvenile parakeets, despite being more infectious than adults, made little impact on *R*_*0*_ estimates. This was due, in part, to the fact that 40% of juveniles were removed by natural mortality from the population by day 135, and the remaining would then “mature” to adults (Table 1). Adult parakeets, which comprised a much larger proportion of the population than juveniles (Table 1), naturally survived longer and had more time to influence ND transmission and *R*_*0*_. As such, lower acute adult morality in model 2 (determined by *c*_*m*_) slightly increased *R*_*0*_ compared to model 1. This suggested that maintaining infected adults for longer time in the population (i.e., not dying from the disease), would exacerbate an ND outbreak even though adults were less infectious compared to juveniles.

We should emphasize that the parameters responsible for the differences in *R*_*0*_ predictions between models 1 and 2 (*c*_*βA*_, *c*_*m*_, and *p*_*1a*_) were uncertain and should be prioritized for future investigation, especially the transmission rate and probability for acutely-infected adult parakeets. However, because model 2 more closely reflects the biological system, it is reasonable to assume that it also more accurately predicts ND dynamics. By extension, it is reasonable to conclude that model 1 overestimated disease transmission and the magnitude of the ND outbreak. In a similar situation, Brooks-Pollock et al. [96] found that more realistic age-specific mortality versus constant mortality rates in mathematical models of human tuberculosis decreased *R*_*0*_ estimates and the effort required for disease control.

### Role of harvest

Through scenario analysis we determined that increasing uncompensated harvest rates (i.e., those not compensated for by natality), had a modest dampening influence on *R*_*0*_, meaning that higher uncompensated harvest increased the probability of ND fadeout (*R*_*0*_ < 1; Table 2). Although the increase in the proportions of fadeout with increased additional harvest was less dramatic in model 2 versus model 1 (6% compared to 12%; Table 2), this increase must be considered along with the already lower *R*_*0*_ estimates in model 2. Compensating additional harvest (*h1*) caused *R*_*0*_ to remain high in both models (> 2.5), indicating that an influx of susceptible individuals (i.e., offspring) may help sustain higher potential for an ND outbreak (Fig 6); however, because the confidence intervals for *R*_*0*_ estimates with compensated and uncompensated harvest largely overlapped (results not shown), largely because of the wide uniform distributions used in our models (Table 1), the influence of host population density-dependent response on *R*_*0*_ should be interpreted cautiously.

Higher harvest rates produced slower population decline in the age-structured population compared to the homogeneous population (Fig 4). This dampened effect in model 2 reflected, in part, the 40% removal of juveniles through natural mortality (Table 1). High juvenile mortality is often exploited in harvested populations because their lower survival rates and lower reproductive value increase the probability of compensation [143]. For this reason, Choisy & Rohani [13] predicted that shifting harvest to younger age classes would decrease the risk of disease-related mortality in hypothetical scenarios, which is supported by our results.

Overall, higher harvest rates had minimal effect on ND dynamics (Fig 3) and population size (Fig 4). This is partly due to the way we prorated the annual harvest rate for the one-day time step in our model simulations, which diluted the effect of harvest, particularly over short time periods such as the 135-day juvenile period. In addition, because we do not know the actual harvest rate, we set our baseline harvest (*h*_*b*_) conservatively and even compensated for it with natality (Table 1). It is likely that even our upper limit of 10% additional annual harvest (*h1*) was conservatively low. In model 2, we fixed the proportion of juvenile (40%) and adult (60%) harvest as the average of what was recorded in market surveys throughout the year. In reality, we know that there are seasonal harvest differences, which would likely influence ND dynamics as they do for other diseases [153,154]. For example in model 2, when we changed the fixed harvest proportions to reflect harvest rates during a nesting period (juveniles 90%; adults 10%), *R*_*0*_ was 20% higher, but the population decline at two years post ND introduction was 12% less compared to the opposite proportions (juveniles 10%, adults 90%; results not shown).

### Key parameters

The duration of the acute (*D*_*δ*_) and chronic (*D*_*γ*_) infectious stages were most influential in determining *R*_*0*,_ as identified in both the univariate correlation (Fig 7) and multivariable classification-tree analyses (Fig 8). The positive Spearman correlations indicated that longer duration of the infectious stages resulted in higher *R*_*0*_ estimates. In poultry operations, decreasing the duration of infectious stages (i.e., the length of time of ND viral shedding), is one of the primary goals for improving vaccination programs [145,155]. With captive pet birds, preventive measures such as vaccination could help diminish clinical signs and the duration and amount of viral shedding. The efficacy of ND vaccination in white-winged parakeets is unknown, although Denadai et al. [112] determined that Australian parakeets could be safely and effectively vaccinated against ND. The negative correlation between the probability of acute disease-related death (*pd*_*A*_) and *R*_*0*_ in both models (Fig 7) reflects the importance of removing acutely-infected individuals from the population. During an outbreak, this could be achieved by quarantining or culling acutely-infected individuals [95]. During outbreaks of ND in commercial flocks, quickly culling all infected and potentially-infected individuals has been a critical component of typical management strategies to prevent expansion of the outbreak [156]. Neither vaccination nor quarantine, however, would be feasible to control ND in wild populations of white-winged parakeets. This highlights the vulnerability of wild populations.

The classification-tree analyses provided a broad perspective of the key parameters and their interactions to produce disease-free or endemic ND states (Fig 8). In addition to the duration of the acute (*D*_*δ*_) and chronic (*D*_*γ*_) infectious stages, the most optimal classification trees identified the transmission rate for chronically-infected parakeets (*β*_*c*_) as influencing *R*_*0*_, rather than the transmission rate for acutely-infected parakeets (*β*_*a*_) as in our correlation analysis. When the interaction of transmission rates was evaluated along with other parameters, it became clear that the rate of virus transmission from chronically-infected birds (*β*_*c*_) to susceptible individuals would become critical in determining whether ND would die out or persist, specifically when chronic infection period (*D*_*γ*_) lasted longer than approximately 47 days (Fig 8). Such insight was impossible evaluating correlation alone, and demonstrates the value of multivariable analyses. Even though the most optimal classification trees retained only three or four parameters, the predictive error rates were relatively low for both models, indicating that the identified optimal classification trees correctly predicted infection fade out or an endemic state in almost 90% of independent simulations.

### Limitations

Our findings are dependent on several modeling assumptions. For instance, we assumed that ND transmission was density dependent in white-winged parakeets. Density-dependent transmission is commonly assumed for wildlife diseases [103,157]. In most cases of wildlife diseases, empirical data are difficult to obtain to confirm transmission, but Hochachka & Dhondt [140] used pre- and post-enzootic data to conclude that mycoplasma conjunctivitis transmission in house finches was density dependent. In some situations, the mode of pathogen transmission may not be constant throughout the year as demographic seasonal traits affect social behavior and spatial structure of the host population [158]. For example, Hosseini et al. [154] combined frequency- and density-dependent transmission for *Mycoplasma gallisepticum* to represent seasonal variation in social structure of house finches.

A similar situation could occur with ND transmission in white-winged parakeets as flock size fluctuates throughout the year [109]. During the roughly 4-month breeding season, when pairs separate for nesting and flock size decreases, transmission may be more consistent with frequency dependence. During the post-breeding period, when adults along with their fledglings rejoin the flock, transmission is more likely to be density dependent. Addressing the role of chance in pathogen transmission could be evaluated by incorporating demographic stochasticity into the model. A stochastic approach could also assess the influence of changing the initial conditions, (e.g., number of ND-infectious individuals released into the population). For instance, we assumed that just one infectious individual would be introduced into a susceptible population of white-winged parakeets. In reality, it is common for authorities to release dozens to hundreds of potentially exposed individuals confiscated from markets [80]. While this was beyond the scope of the work presented here, including demographic stochasticity and density-dependent population natality and mortality processes in a seasonal age-structured model would provide a method to more thoroughly investigate the influence of harvest on ND dynamics [151].

### Recommendations

Few realistic options exist to control an ND outbreak in wild white-winged parakeets or other parrots. Assuring that *criollo* chickens and fighting cocks in the Amazonian region are vaccinated against ND would reduce the probability of cross infection to psittacine species in animal markets. In our models, we used a non-threatened psittacine species; however, many parrots are threatened in Peru—often by illegal trade—and could be seriously affected by an introduced infectious disease [46]. The most effective preventive measure would be to avoid releasing confiscated parakeets without prior health screening and, ideally, preventing illegal harvest in the first place. Authorities should coordinate more effectively with non-governmental organizations in Peru working to decrease the illegal wildlife trade including wildlife rescue centers and zoological parks [54], to assist with quarantine and rehabilitation of confiscated individuals. Similarly, authorities could increase collaboration with veterinary colleges to assist with physical exams and diagnostic testing of confiscated animals. Finally, combined efforts to increase enforcement of Peru’s wildlife legislation and to decrease demand for wild-caught native birds for the domestic pet market would help mitigate the risk of introducing infectious diseases.

In conclusion, our study improved understanding of ND dynamics in a wild population of harvested psittacines. We demonstrated that the hypothetical release of a confiscated individual infected with ND would provoke considerable population decline in a wild population of white-winged parakeets. To our knowledge, this is the first study to use infectious disease modeling to link illegal wildlife trade and disease introduction in a native wildlife population. The differences we observed in both *R*_*0*_ and disease dynamics between our homogeneous and age-structured populations highlight the importance of incorporating even simplistic age structure in disease modeling. While we recognize that further enhancements, such as including density-dependent regulation and demographic stochasticity, could contribute to the understanding of ND dynamics, our initial models provide a baseline for future evaluation. We encourage the conservation community to examine other disease risks associated with illegal wildlife trade, particularly in endangered species where disease may contribute to species extinctions.

## Supporting Information

S1 Fig Deterministic two-year time trajectories for Newcastle disease transmission for juvenile and adult white-winged parakeets. (PDF)

S2 Fig Comparison of the effect of fixed and uncertain population size on population size. (PDF)

S3 Fig Comparison of the effect of fixed and uncertain population size on *R*_*0*_. (PDF)

S4 Fig Mean basic reproductive number (*R*_*0*_) estimates. (PDF)

S5 Fig Population size following Newcastle disease (ND) introduction. (PDF)

S1 File Matlab codes for Model 1 and Model 2. (PDF)

S2 File Analytic derivation of the basic reproduction number (*R*_*0*_). (PDF)

S3 File Photographs of parrots and chickens at animal markets in three Peruvian cities. (PDF)
