# Supplementary material for: Interacting Effects of Newcastle Disease Transmission and Illegal Trade on a Wild Population of White-Winged Parakeets in Peru: A Modeling Approach
Source: PLoS One. 2016 Jan 27;11(1):e0147517. doi: 10.1371/journal.pone.0147517 (PMC4731398; doi:10.1371/journal.pone.0147517)
Supplement: S1 Fig — (PDF) [file pone.0147517.s001.pdf]

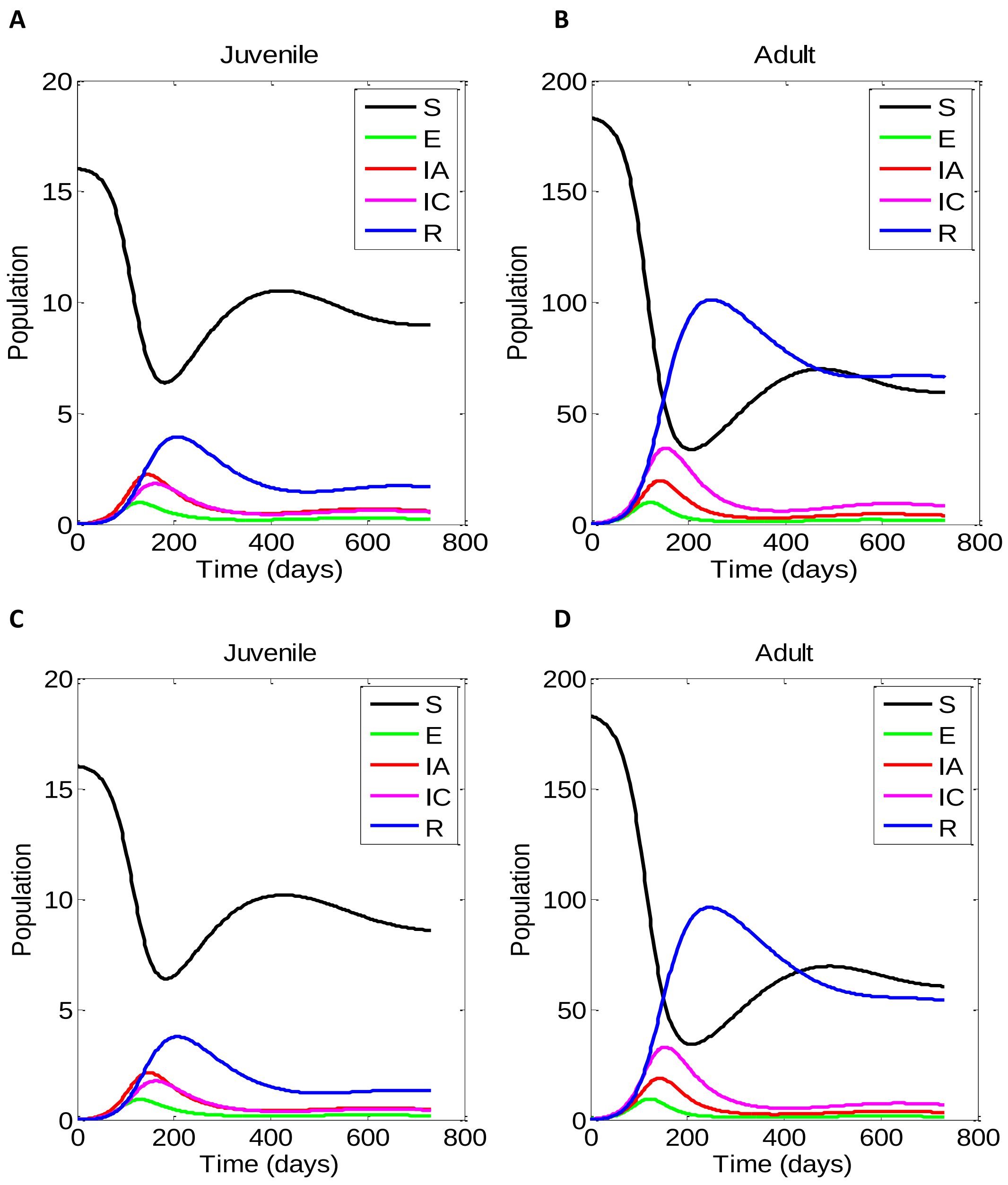

S1 Figure. Deterministic two-year time trajectories for Newcastle disease transmission for juvenile and adult white-winged parakeets.

Model 2 with (A–B) no additional harvest (*hl* = 0) and (C–D) 10% additional (uncompensated) harvest (*hl* = 10%). Depicted are susceptible (*S*), exposed (*E*), acutely-infected (*IA*), chronically-infected (*IC*) and recovered (*R*) states for juveniles and adults.
