# Supplementary material for: Interacting Effects of Newcastle Disease Transmission and Illegal Trade on a Wild Population of White-Winged Parakeets in Peru: A Modeling Approach
Source: PLoS One. 2016 Jan 27;11(1):e0147517. doi: 10.1371/journal.pone.0147517 (PMC4731398; doi:10.1371/journal.pone.0147517)
Supplement: S2 Fig — (PDF) [file pone.0147517.s002.pdf]

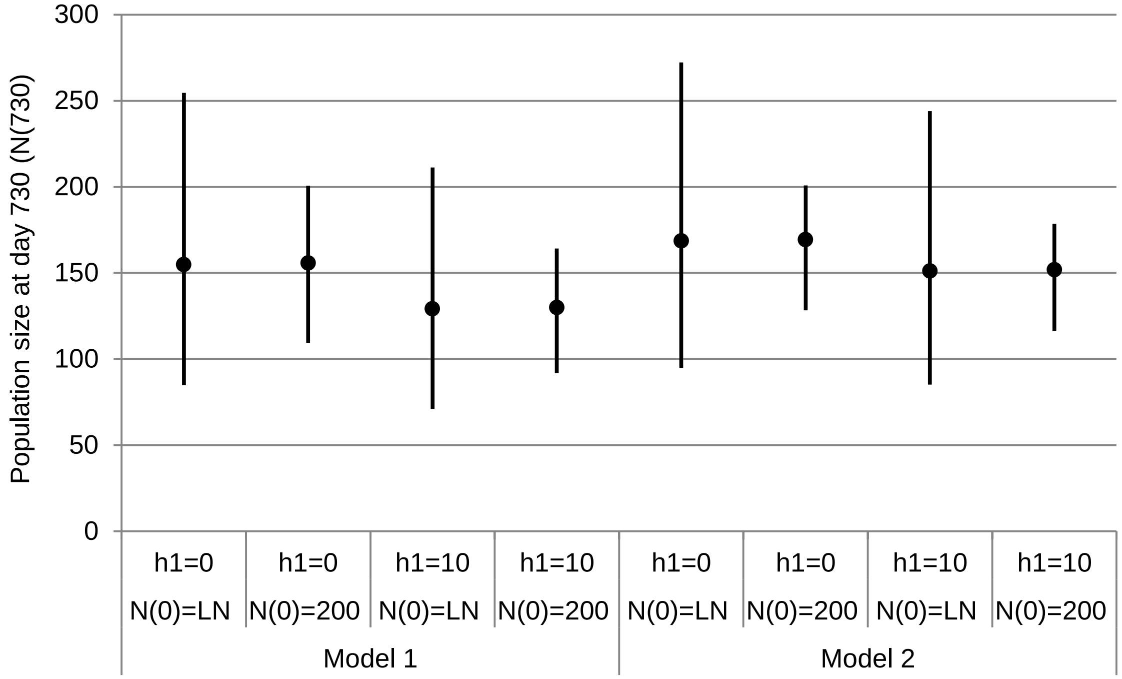

S2 Figure. Comparison of the effect of a fixed and uncertain initial population size on the predicted population size at two years post-introduction of Newcastle disease into a homogeneous (model 1) and age-structured (model 2) population of white-winged parakeets. Black dots show the mean predicted population size while the bars indicate the associated confidence intervals (based on 5th and 95th percentiles). The initial population size was either fixed (N(0)=200) or uncertain (N(0)=LN where LN=Lognormal (189, 1.4)), while additional harvest was either (h1=0) or 10% (h1=10). For each scenario, results were based on 10,000 simulations.
