# Supplementary material for: Interacting Effects of Newcastle Disease Transmission and Illegal Trade on a Wild Population of White-Winged Parakeets in Peru: A Modeling Approach
Source: PLoS One. 2016 Jan 27;11(1):e0147517. doi: 10.1371/journal.pone.0147517 (PMC4731398; doi:10.1371/journal.pone.0147517)
Supplement: S3 Fig — (PDF) [file pone.0147517.s003.pdf]

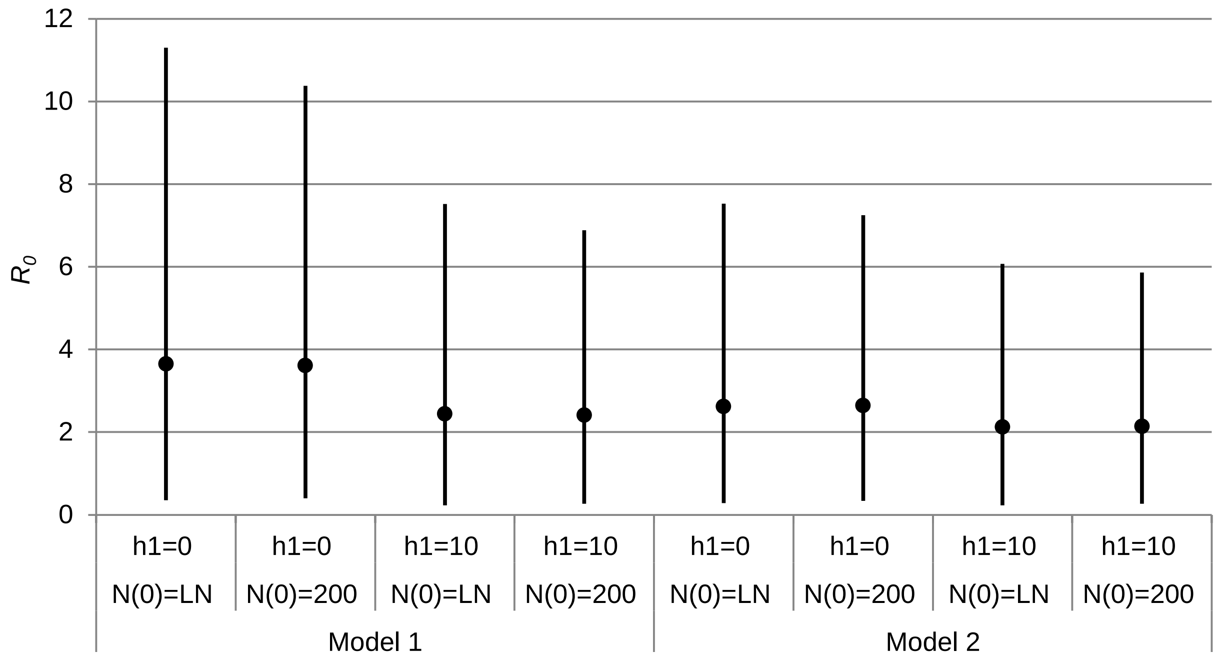

S3 Figure. Comparison of the effect of a fixed and uncertain initial population size on R_0 for Newcastle disease in a homogeneous (model 1) and age-structured (model 2) population of white-winged parakeets. Black dots show the mean R_0 while the bars indicate the associated confidence intervals (based on 5th and 95th percentiles). The initial population size was either fixed (N(0)=200) or uncertain (N(0)=LN where LN=Lognormal (189, 1.4)), while additional harvest was either absent (h1=0) or 10% (h1=10). For each scenario, results were based on 10,000 simulations.
