# Supplementary material for: Interacting Effects of Newcastle Disease Transmission and Illegal Trade on a Wild Population of White-Winged Parakeets in Peru: A Modeling Approach
Source: PLoS One. 2016 Jan 27;11(1):e0147517. doi: 10.1371/journal.pone.0147517 (PMC4731398; doi:10.1371/journal.pone.0147517)
Supplement: S4 Fig — (PDF) [file pone.0147517.s004.pdf]

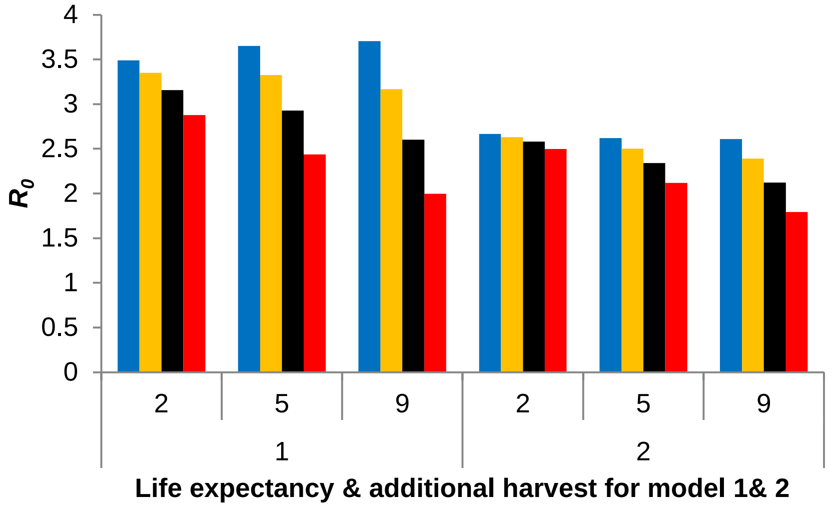

S4 Figure. Mean basic reproductive number (R_0) estimates.

Mean R_0 estimates from 10,000 simulations of Newcastle disease transmissions for homogenous (model 1) and age-structure (model 2) populations of white-winged parakeets with no additional harvest (*h1*=0 blue) and three additional uncompensated harvest rates (*h1*=2% orange, *h1*=5% black, and *h1*=10% red) and for three life expectancies (D_d = two, five, and nine years).
