# Supplementary material for: Interacting Effects of Newcastle Disease Transmission and Illegal Trade on a Wild Population of White-Winged Parakeets in Peru: A Modeling Approach
Source: PLoS One. 2016 Jan 27;11(1):e0147517. doi: 10.1371/journal.pone.0147517 (PMC4731398; doi:10.1371/journal.pone.0147517)
Supplement: S1 File — (PDF) [file pone.0147517.s006.pdf]

S1 File. Mathematical codes for Model 1 and Model 2.

Model 1

```
% This function defines the differential equations for an epidemic model of Newcastle Disease
transmission in a homogeneous population of white-winged parakeets.

function dx=Newcastle3(t,x)

% Parameters
N=200;
d=1/(5.0*365);  % Natural mortality rate; tested at 2, 5 and 9 years
h=0.01/365;  % Total harvest rate
h1=0;  % Additional harvest rate; tested at 0, 2, 5, 10%
betaA=3/(14*N);  % Transmission rate (Acute-to-Susceptible)
Ir=0.1;  % Infectiousness reduction coefficient for chronically-infected parakeets
betaC=Ir*betaA;  % Transmission rate (Chronic-to-Susceptible)
Dalph=5.5;  % Duration of latency
alpha=1/Dalph;  % Rate of becoming infectious
Ddelt=30;  % Duration of acute infection
delta=1/Ddelt;  % Rate of leaving acutely infectious stage
Dgam=39;  % Duration of chronic infection
gamma=1/Dgam;  % Recovery rate for chronic infection
muA=0.25*delta;  % Disease-related mortality rate (acute)
muC=0.075*gamma;  % Disease-related mortality rate (chronic)
p1=0.625;  % Probability of acute infection
p2=0.5;  % Probability of recovery from acute infection
Dnu=243;  % Duration of immunity
nu=1/Dnu;  % Loss of immunity rate

% ODEs
dx=zeros(5,1);
dx(1)=(d+h)*sum(x)-(d+h+h1)*x(1)-betaA*x(1)*x(3)-betaC*x(1)*x(4)+nu*x(5);
dx(2)=betaA*x(1)*x(3)+betaC*x(1)*x(4)-(d+h+h1+alpha)*x(2);
dx(3)=alpha*p1*x(2)-(d+h+h1+muA+delta)*x(3);
dx(4)=alpha*(1-p1)*x(2)+delta*(1-p2)*x(3)-(d+h+h1+muC+gamma)*x(4);
dx(5)=delta*p2*x(3)+gamma*x(4)-(d+h+h1+nu)*x(5);
```

Model 2

% This function defines the differential equations for an epidemic model of Newcastle Disease transmission in an age-structured population of white-winged parakeets.

```
function dx=NewcastleAge4(t,x)

% Parameters
N=200;
h=0.01/365;  % Total harvest rate
h1=0;  % Additional harvest rate; tested at 0, 2, 5, and 10%
DOmeg=135;  % Duration of juvenile stage
Omega=1/DOmeg;
Ddj=-DOmeg/log(0.6);  % Juvenile life expectancy; tested at 0.5, 0.6, and 0.7%
dj=1/Ddj;  % Natural juvenile mortality rate
Dd=5.0*365;  % Life expectancy; tested at 2, 5, and 9 years
Dda=Dd-Ddj;  % Adult life expectancy
da=1/Dda;  % Adult death rate
hj=0.4*(h+h1);  % Total juvenile harvest rate
ha=0.6*(h+h1);  % Total adult harvest rate
hjl=0.4*h;  % Juvenile harvest rate for lambda
hal=0.6*h;  % Adult harvesting rate for lambda
betaA=3/(14*N);  % Transmission rate (Acute-to-Susceptible)
Ir=0.1;  % Infectiousness reduction coefficient for chronically-infected parakeets
cbetaa=0.25;  % Age-dependent acute transmission factor
beta1=(1+cbetaa)*betaA;  % Transmission rate (Acute-Juvenile-to-Susceptible)
beta2=Ir*betaA;  % Transmission rate (Chronic-Juvenile-to-Susceptible)
beta3=(1-cbetaa)*betaA;  % Transmission rate (Acute-Adult-to-Susceptible)
beta4=Ir*betaA;  % Transmission rate (Chronic-Adult-to-Susceptible)
beta5=beta3;
beta6=beta4;
beta7=beta1;
beta8=beta2;
Dalph=5.5;  % Duration of latency
alpha=1/Dalph;
Ddelt=30;  % Duration of acute infection
delta=1/Ddelt;
Dgam=39;  % Duration of chronic infection
gamma=1/Dgam;
Dnu=243;  % Duration of immunity
nu=1/Dnu;
muA=0.25*delta;  % Disease-related mortality rate (acute)
muC=0.075*gamma;  % Disease-related mortality rate (chronic)
```

```
cm=0.25;  % Age-dependent acute mortality factor
muAj=(1+cm)*muA;  % Disease-related mortality rate for acutely-infected juvenile parakeet
muCj=muC;  % Disease-related mortality rate for chronically-infected juvenile parakeet
muAa=(1-cm)*muA;  % Disease-related mortality rate for acutely-infected adult parakeet
muCa=muC;  % Disease-related mortality rate for chronically-infected adult parakeet
p1j=0.75;  % Probability of acute juvenile infection
p1a=0.5;  % Probability of acute adult infection
p2=0.5;  % Probability of recovery from acute infection

% ODEs
dx=zeros(10,1);

% Juveniles
dx(1)=(dj+hjl+Omega)*(da+hal)*sum(x)/(da+hal+Omega)-(dj+hj+Omega)*x(1)-beta1*x(1)*x(3)-
beta2*x(1)*x(4)-beta3*x(1)*x(8)-beta4*x(1)*x(9)+nu*x(5);
dx(2)=beta1*x(1)*x(3)+beta2*x(1)*x(4)+beta3*x(1)*x(8)+beta4*x(1)*x(9)-(dj+hj+Omega+alpha)*x(2);
dx(3)=alpha*p1j*x(2)-(dj+hj+Omega+muAj+delta)*x(3);
dx(4)=alpha*(1-p1j)*x(2)+delta*(1-p2)*x(3)-(dj+hj+Omega+muCj+gamma)*x(4);
dx(5)=delta*p2*x(3)+gamma*x(4)-(dj+hj+Omega+nu)*x(5);

% Adults
dx(6)=Omega*x(1)-(da+ha)*x(6)-beta5*x(6)*x(8)-beta6*x(6)*x(9)-beta7*x(6)*x(3)-
beta8*x(6)*x(4)+nu*x(10);
dx(7)=Omega*x(2)+beta5*x(6)*x(8)+beta6*x(6)*x(9)+beta7*x(6)*x(3)+beta8*x(6)*x(4)-
(da+ha+alpha)*x(7);
dx(8)=Omega*x(3)+alpha*p1a*x(7)-(da+ha+muAa+delta)*x(8);
dx(9)=Omega*x(4)+alpha*(1-p1a)*x(7)+delta*(1-p2)*x(8)-(da+ha+muCa+gamma)*x(9);
dx(10)=Omega*x(5)+delta*p2*x(8)+gamma*x(9)-(da+ha+nu)*x(10);
```
