# Supplementary material for: Interacting Effects of Newcastle Disease Transmission and Illegal Trade on a Wild Population of White-Winged Parakeets in Peru: A Modeling Approach
Source: PLoS One. 2016 Jan 27;11(1):e0147517. doi: 10.1371/journal.pone.0147517 (PMC4731398; doi:10.1371/journal.pone.0147517)
Supplement: S2 File — (PDF) [file pone.0147517.s007.pdf]

S2 File. Derivation of the basic reproduction number (*R_0*) using the next-generation matrix method.

The expressions for the basic reproduction number (R_0) were derived using the next-generation matrix (NGM) method originally defined by van den Driessche & Watmough [1]. It is straightforward to verify that models 1 and 2 satisfy the conditions (A1)—(A5) in [1] needed to apply this method. Indeed, (A1) the directed transfer of individuals between compartments can be expressed as the difference between positive-valued functions *Fi* and *Vi*, *Fi* - *Vi*. (A2) If a compartment is empty, then there can be no transfer out of the compartment. (A3) The incidence of infection for uninfected compartments is zero. (A4) There is no immigration of infected individuals into the system. (A5) In the absence of new infections, the disease-free equilibrium (DFE) is stable.

To construct the next-generation matrix, (FV^{-1}), we defined the matrices *F* and *V* as:

$$F = \left[\frac{\partial F_i(x)}{\partial x_j}\right] x = x_0 \text{ and } V = \left[\frac{\partial V_i(x)}{\partial x_j}\right] x = x_0,$$

where the (*i,j*) entry of matrix *F* was the rate at which infected individuals in compartment *j* produce new infections in compartment *i* and the (*i, j*) entry of *V* was the net rate of change of animals in compartment by any other means. We considered that E, I_A and I_C are the infected states but that new infections occurred only in the *E* compartment. The rates were evaluated at the disease-free equilibrium $x = x_0$.

Setting $\vec{I} = (E, I_A, I_C)^T$ for model 1,it follows that

$$\frac{d\vec{I}}{dt} = J\vec{I} = (F - V)\vec{I},$$

where *J* denotes the Jacobian matrix evaluated at the DFE and *F* and *V* matrices are:

$$F = \begin{bmatrix} 0 & \beta_A S0 & \beta_C S0 \\ 0 & 0 & 0 \\ 0 & 0 & 0 \end{bmatrix}$$

$$V = \begin{bmatrix} d + h + \alpha & 0 & 0 \\ -\alpha p_1 & d + h + \mu_A + \delta & 0 \\ -\alpha(1 - p_1) & -\delta(1 - p_2) & d + h + \mu_C + \gamma \end{bmatrix}$$

Following [1,2], the basic reproduction number R_0 is defined as the spectral radius (dominant eigenvalue) of matrix FV^{-1}. That is,

$$R_0 = \rho(FV^{-1}) = R_{0A} + R_{0C}, \quad \text{(A1)}$$

where

$$R_{0A} = \frac{\alpha p_1 \beta_A S0}{(d + h + \alpha)(d + h + \mu_A + \delta)}$$

$$R_{0C} = \left[\frac{\alpha(1 - p_1)}{(d + h + \alpha)} + \frac{\alpha p_1 \delta(1 - p_2)}{(d + h + \alpha)(d + h + \mu_A + \delta)}\right] \frac{\beta_C S0}{(d + h + \mu_C + \gamma)}$$

The expression for R_{0A} can be interpreted as follows: a fraction $\alpha p1/(d + h + \alpha)$ of exposed hosts E progress to state I_A and spend an average of $1/(d + h + \mu_A + \delta)$ days in state I_A over the expected duration of infection. Multiplying by $\beta_A S0$ gives the expected number of secondary infections resulting from interactions between susceptible and acutely-infected hosts.

Similarly, the expression for R_{0C} can be interpreted as follows: a fraction $\alpha p_1/(d+h+\alpha)$ of exposed hosts E progress to state I_A, and of these acutely- infected hosts, a fraction $\delta(1-p_2)/(d + h + \mu_A + \delta)$ progress to state I_C spending an average of $1/(d + h + \mu_C + \gamma)$ days in state I_C over the expected duration of infection. Alternatively, a fraction $\alpha(1-p_1)/(d+h+\alpha)$ of exposed hosts progress directly to state I_C and spend an average of $1/(d + h + \mu_C + \gamma)$ days in state I_C. Multiplying by $\beta_C S0$ gives the expected number of secondary infections resulting from interactions between susceptible and chronically-infected hosts.

For model 2, we considered that E_j, I_{Aj}, I_{Cj}, E_a, I_{Aa}, and I_{Ca} are the infected states but that new infections occur only when a susceptible bird S_j or S_a became exposed E_j or E_a. Thus, F and V matrices are:

$$F = \begin{bmatrix} 0 & \beta_{Aj} S_{j0} & \beta_C S_{j0} & 0 & \beta_{Aa} S_{j0} & \beta_C S_{j0} \\ 0 & 0 & 0 & 0 & 0 & 0 \\ 0 & 0 & 0 & 0 & 0 & 0 \\ 0 & \beta_{Aj} S_{a0} & \beta_C S_{a0} & 0 & \beta_{Aa} S_{a0} & \beta_C S_{a0} \\ 0 & 0 & 0 & 0 & 0 & 0 \\ 0 & 0 & 0 & 0 & 0 & 0 \end{bmatrix},$$

$$V = \begin{bmatrix} c_1 & 0 & 0 & 0 & 0 & 0 \\ -\alpha p_1 & c_2 & 0 & 0 & 0 & 0 \\ -\alpha(1-p_1) & -\delta(1-p_1) & c_3 & 0 & 0 & 0 \\ -\Omega & 0 & 0 & c_4 & 0 & 0 \\ 0 & -\Omega & 0 & -\alpha p_1 & c_5 & 0 \\ 0 & 0 & -\Omega & -\delta(1-p_1) & -\delta(1-p_2) & c_6 \end{bmatrix},$$

where
$c_1 = d_j + h_j + \Omega + \alpha,$
$c_2 = d_j + h_j + \Omega + \mu_{Aj} + \delta,$
$c_3 = d_j + h_j + \Omega + \mu_{Cj} + \gamma,$
$c_4 = d_a + h_a + \alpha,$
$c_5 = d_a + h_a + \mu_{Aa} + \delta,$
$c_6 = d_a + h_a + \mu_{Ca} + \gamma.$

Thus, R_0 is defined as:

$$R_0 = \rho(FV^{-1}) = ½ \left[R_{01} + R_{02} + \sqrt{(R_{01} - R_{02})^2 + 4R_{03}R_{04}}\right], \qquad \text{(A2)}$$

where

$$R_{01} = \frac{\alpha p_1 \beta_{Aj} S_{j0}}{c_1c_2} + \frac{\alpha(1-p_1)\beta_C S_{j0}}{c_1c_3} + \frac{\alpha p_1 \delta(1-p_2)\beta_C S_{j0}}{c_1c_2c_3} + \frac{\Omega\alpha p_1 \beta_{Aa} S_{j0}}{c_1c_4c_5} + \frac{\Omega\alpha p_1 \beta_{Aa} S_{j0}}{c_1c_2c_5} + \frac{\Omega\alpha(1-p_1)\beta_C S_{j0}}{c_1c_4c_6} + \frac{\Omega\alpha(1-p_1)\beta_C S_{j0}}{c_1c_3c_6} + \frac{\Omega\alpha p_1 \delta(1-p_2)\beta_C S_{j0}}{c_1c_4c_5c_6} + \frac{\Omega\alpha p_1 \delta(1-p_2)\beta_C S_{j0}}{c_1c_2c_5c_6} + \frac{\Omega\alpha p_1 \delta(1-p_2)\beta_C S_{j0}}{c_1c_2c_3c_6},$$

$$R_{02} = \frac{\alpha p_1 \beta_{Aj} S_{a0}}{c_1c_2} + \frac{\alpha(1-p_1)\beta_C S_{a0}}{c_1c_3} + \frac{\alpha p_1 \delta(1-p_2)\beta_C S_{a0}}{c_1c_2c_3} + \frac{\Omega\alpha p_1 \beta_{Aa} S_{a0}}{c_1c_4c_5} + \frac{\Omega\alpha p_1 \beta_{Aa} S_{a0}}{c_1c_2c_5} + \frac{\Omega\alpha(1-p_1)\beta_C S_{a0}}{c_1c_4c_6} + \frac{\Omega\alpha(1-p_1)\beta_C S_{a0}}{c_1c_3c_6} + \frac{\Omega\alpha p_1 \delta(1-p_2)\beta_C S_{a0}}{c_1c_4c_5c_6} + \frac{\Omega\alpha p_1 \delta(1-p_2)\beta_C S_{a0}}{c_1c_2c_5c_6} + \frac{\Omega\alpha p_1 \delta(1-p_2)\beta_C S_{a0}}{c_1c_2c_3c_6},$$

$$R_{03} = \frac{\alpha p_1 \beta_{Aa} S_{j0}}{c_4c_5} + \frac{\alpha(1-p_1)\beta_C S_{j0}}{c_4c_6} + \frac{\alpha p_1 \delta(1-p_2)\beta_C S_{j0}}{c_4c_5c_6},$$

$$R_{04} = \frac{\alpha p_1 \beta_{Aa} S_{a0}}{c_4c_5} + \frac{\alpha(1-p_1)\beta_C S_{a0}}{c_4c_6} + \frac{\alpha p_1 \delta(1-p_2)\beta_C S_{a0}}{c_4c_5c_6}.$$

The terms R_{01} and R_{02} represent the average number of secondary juvenile or adult infections, respectively, produced by one exposed juvenile E_j during its entire infectious period. The terms R_{03} and R_{04} represent the average number of secondary juvenile or adult infections produced by one exposed adult E_a during its entire infectious period, respectively.

References

1. van den Driessche P, Watmough J (2002) Reproduction numbers and sub-threshold endemic equilibria for compartmental models of disease transmission. Mathematical Biosciences 180: 29-48.

2. Diekmann O, Heesterbeek JAP, Metz JAJ (1990) On the definition and the computation of the basic reproduction ratio (R0) in models for infectious diseases in heterogeneous populations. Journal of Mathematical Biology 28: 365-382.
