# Supplementary material for: Interacting Effects of Newcastle Disease Transmission and Illegal Trade on a Wild Population of White-Winged Parakeets in Peru: A Modeling Approach
Source: PLoS One. 2016 Jan 27;11(1):e0147517. doi: 10.1371/journal.pone.0147517 (PMC4731398; doi:10.1371/journal.pone.0147517)

S3 File. Photographs taken at animal markets in three Peruvian cities (A. Tumbes; B. Pucallpa; C. Lima) demonstrating numerous native parrot species (red circles), including white-winged parakeets (white circles), and chickens (blue circles) offered for sale in close proximity.

A.

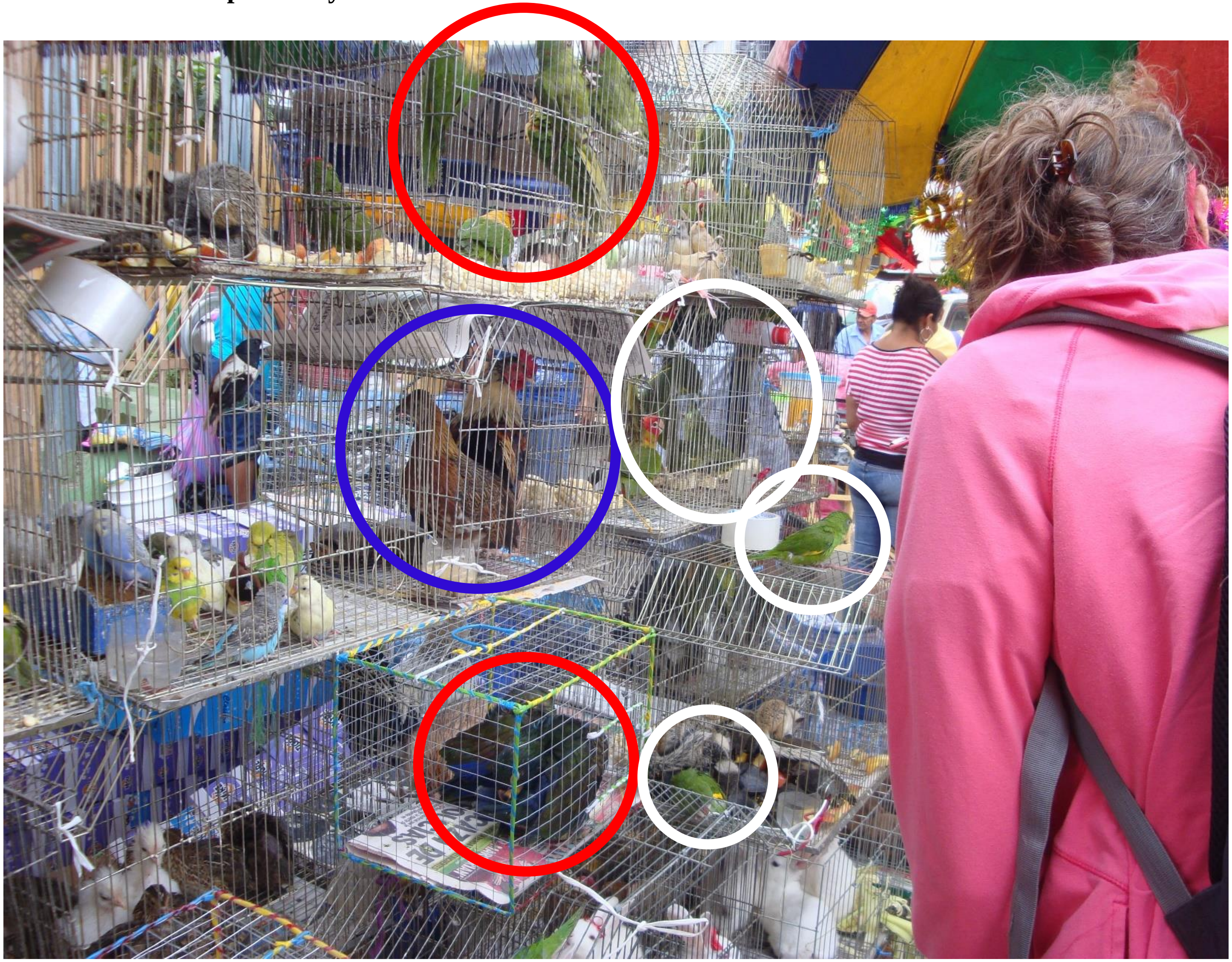

B.

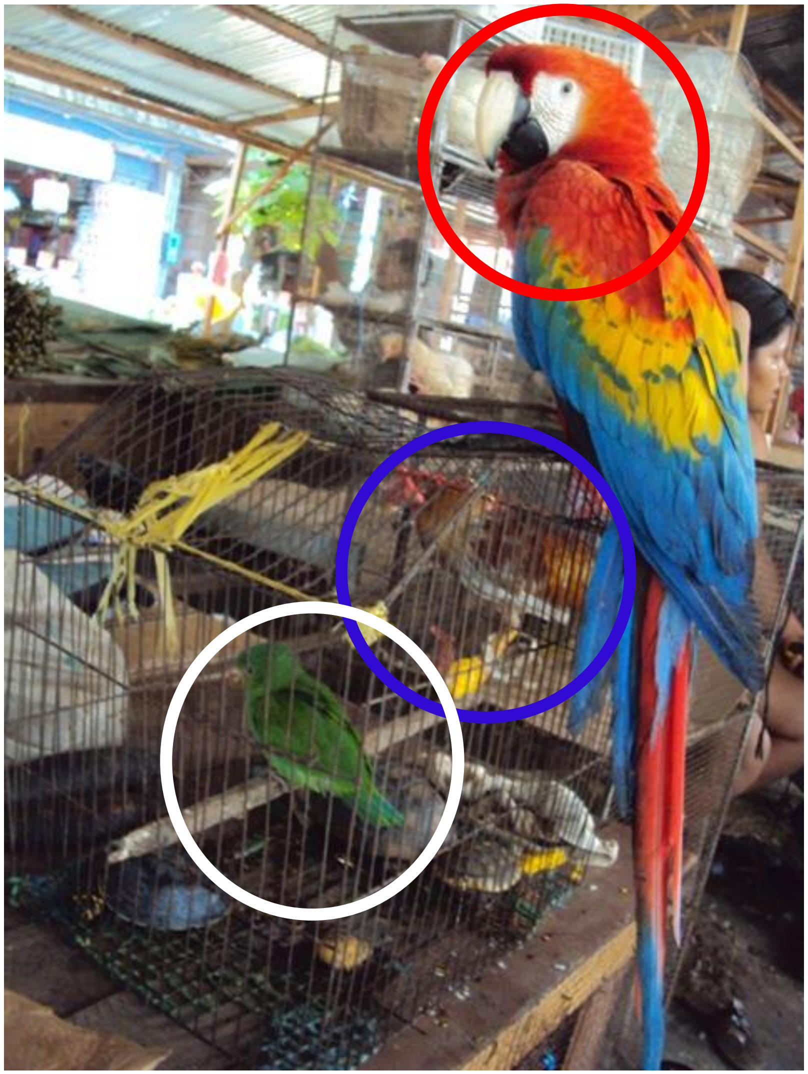

Photo by Neotropical Primate Conservation

C.

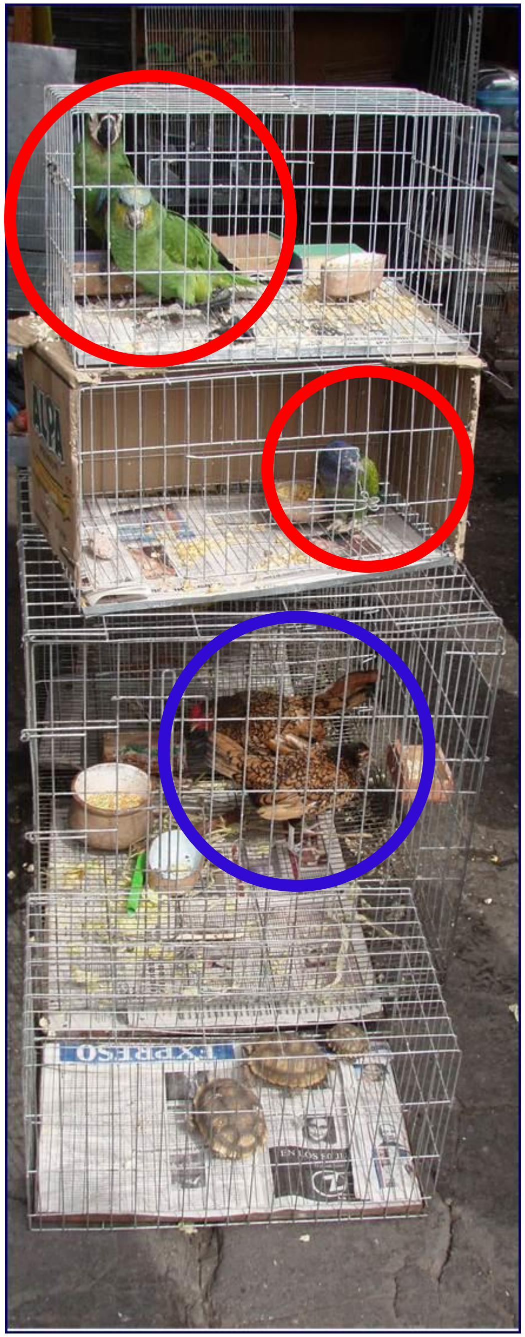

Supplement: S3 File — (PDF) [file pone.0147517.s008.pdf]
